# A novel carotenoid cleavage activity involved in the biosynthesis of *Citrus* fruit-specific apocarotenoid pigments

**DOI:** 10.1093/jxb/ert260

**Published:** 2013-09-04

**Authors:** María J. Rodrigo, Berta Alquézar, Enriqueta Alós, Víctor Medina, Lourdes Carmona, Mark Bruno, Salim Al-Babili, Lorenzo Zacarías

**Affiliations:** ^1^Instituto de Agroquímica y Tecnología de Alimentos, Consejo Superior de Investigaciones Científicas (IATA-CSIC), Av. Agustín Escardino 7, 46980 Paterna, Valencia, Spain; ^2^Faculty of Biology, University of Freiburg, D-79104 Freiburg, Germany

**Keywords:** Apocarotenoid, carotenoid cleavage dioxygenase, carotenoid, β-citraurin, *Citrus*, fruit coloration.

## Abstract

*Citrus* is the first tree crop in terms of fruit production. The colour of *Citrus* fruit is one of the main quality attributes, caused by the accumulation of carotenoids and their derivative C_30_ apocarotenoids, mainly β-citraurin (3-hydroxy-β-apo-8′-carotenal), which provide an attractive orange-reddish tint to the peel of oranges and mandarins. Though carotenoid biosynthesis and its regulation have been extensively studied in *Citrus* fruits, little is known about the formation of C_30_ apocarotenoids. The aim of this study was to the identify carotenoid cleavage enzyme(s) [CCD(s)] involved in the peel-specific C_30_ apocarotenoids. *In silico* data mining revealed a new family of five *CCD4-type* genes in *Citrus*. One gene of this family, *CCD4b1*, was expressed in reproductive and vegetative tissues of different *Citrus* species in a pattern correlating with the accumulation of C_30_ apocarotenoids. Moreover, developmental processes and treatments which alter *Citrus* fruit peel pigmentation led to changes of β-citraurin content and *CCD4b1* transcript levels. These results point to the involvement of CCD4b1 in β-citraurin formation and indicate that the accumulation of this compound is determined by the availability of the presumed precursors zeaxanthin and β-cryptoxanthin. Functional analysis of CCD4b1 by *in vitro* assays unequivocally demonstrated the asymmetric cleavage activity at the 7′,8′ double bond in zeaxanthin and β-cryptoxanthin, confirming its role in C_30_ apocarotenoid biosynthesis. Thus, a novel plant carotenoid cleavage activity targeting the 7′,8′ double bond of cyclic C_40_ carotenoids has been identified. These results suggest that the presented enzyme is responsible for the biosynthesis of C_30_ apocarotenoids in *Citrus* which are key pigments in fruit coloration.

## Introduction

*Citrus* fruit is the first tree crop in the world in terms of production and also because both fresh fruit and juice products are highly consumed and demanded worldwide (FAO, 2012). For the fresh market, the external colour of *Citrus* fruit is a crucial quality feature, and uniform and attractive bright orange coloration in orange, tangerine, and mandarin is probably the main factor determining consumer acceptance. External and internal coloration of *Citrus* fruits, as in many other fruits, is due to the accumulation of carotenoids, which provide colours ranging from yellow and orange to the red tint typical of the different species and cultivars (reviewed in Alquézar *et al.*, 2008*a*; Kato, 2012).

Carotenoids are a diverse group of C_40_ isoprenoid pigments synthesized and accumulated in plastids. The first committed step in carotenoid biosynthesis (for reviews, see Hirschberg, 2001; Fraser and Bramley, 2004; DellaPenna and Pogson, 2006; Cazzonelli, 2011) is mediated by the phytoene synthase that catalyses the condensation of two geranylgeranyl-diphospahate (C_20_) molecules, yielding the colourless carotenoid phytoene (C_40_). Phytoene is then converted into the red all-*trans*-lycopene in a series of reactions extending the conjugated double bond system and involving the enzymes phytoene desaturase, ζ-carotene desaturase, and at least two *cis*-*trans*-isomerases (Isaacson *et al.*, 2002; Park *et al.*, 2002; Chen *et al.*, 2010). From all-*trans*-lycopene, the pathway branches into β-carotene (β,β carotene) formed by lycopene-β-cyclase, and xanthophylls derived from, for example, β-cryptoxanthin, zeaxanthin, and violaxanthin, and α-carotene (β,ε carotene), formed by the combined activity of lycopene-β-cyclase and lycopene-ε-cyclase, and its derivative the xanthophyll lutein. Carotenoids are essential components for photosynthesis, photoprotection, and as precursors of the phytohormones abscisic acid (ABA), strigolactones, and other signalling molecules (Cazzonelli, 2011). Moreover, this group of pigments provides the characteristic bright coloration to numerous fruits, flowers, and storage plant organs, which attracts pollinators and seed dispersal animals (Hirschberg, 2001; Fraser and Bramley, 2004). In animals, carotenoids are of great dietary importance, since some of them have antioxidant activity and are the precursors of vitamin A, and also because they have important ecophysiological functions as pigments (Britton, 1998*a*; Blount and McGraw, 2008). Moreover, consumption of carotenoid-rich foods can provide important health benefits, as some carotenoids have been shown to reduce the incidence of cardiovascular problems, cancer, and other chronic and degenerative diseases (Fraser and Bramley, 2004; Rao and Rao, 2007).

In the last decade, due to the relevance of carotenoids in *Citrus* fruits, important efforts have been made to understand the biochemical and molecular bases for the regulation of carotenoid biosynthesis and accumulation in the diversity of fruits of this genus (Alquézar *et al.*, 2008*a*; Kato *et al*., 2012). Thus, it is known that the peel of immature *Citrus* fruit displays a carotenoid profile characteristic of chloroplast-containing tissues, with lutein as the main carotenoid. At the onset of fruit coloration, lutein becomes gradually replaced by specific β,β-xanthophylls such as 9-*Z*-violaxanthin in the peel and pulp of mature orange fruit, and β-cryptoxanthin and 9-*Z*-violaxanthin in mandarins (Kato *et al.*, 2004). These changes in carotenoid content and composition during ripening of *Citrus* fruit are accompanied by an increase of the transcript level of *phytoene synthase* (*PSY*), *phytoene desaturase* (*PDS*), *ζ-carotene desaturase* (*ZDS*), and *β-carotene hydroxylase* (*β-CHX*) genes (Kato *et al.*, 2004; Rodrigo *et al.*, 2004). The shift from the ε,β-branch to the β,β-branch of the pathway is also coordinated with a decrease in the transcript level of the lycopene ε-cyclase (*ε-LCY*) gene and an up-regulation of a chromoplast-specific lycopene β-cyclase (*β-LCY2*) gene (Kato *et al.*, 2004; Rodrigo *et al.*, 2004; Alquézar *et al.*, 2009). Taken together, these results suggest that carotenoid accumulation and composition during *Citrus* fruit maturation is highly regulated by coordinated transcriptional changes in carotenoid biosynthetic genes, and cyclization of lycopene appears to be a key regulatory step in the pathway, redirecting the flow of carotenoids during the transition from chloroplast to chromoplast (Alquézar *et al.*, 2009; Kato, 2012; Zhang *et al.*, 2012).

**Fig. 1.**
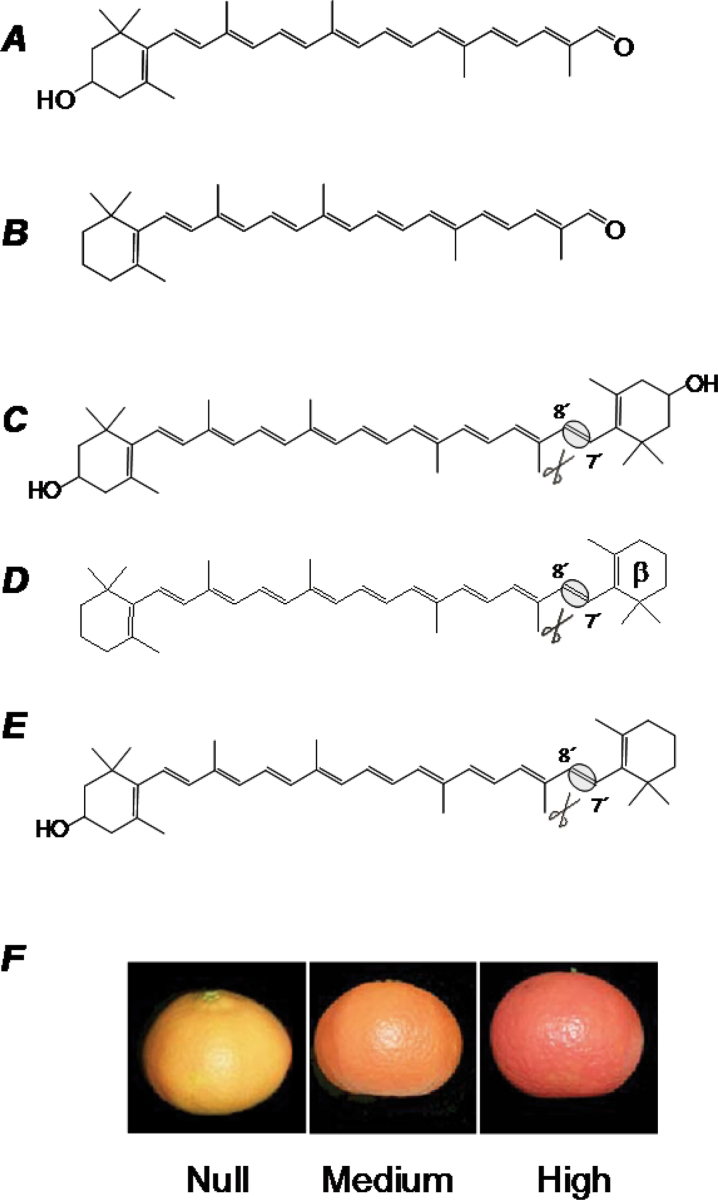
Structure of the main C_30_ apocarotenoids identified in citrus fruits (A, β-citraurin; B, β-apo-8′-carotenal) and the potential *in vivo* precursors (C, zeaxanthin; D, β-carotene; E, β-cryptoxanthin). The asymmetric cleavage site at the 7′,8′ double bonds is indicated. In (F), the different coloration of three different mandarin fruits with a similar C_40_ carotenoid composition in the peel but displaying marked difference in the C_30_ apocarotenoid β-citraurin content is shown: null content, a clementine mutant (39E7, Rios *et al.*, 2010); medium content, a clementine (Gross, 1987; Rios *et al.*, 2010); high content, a mandarin hybrid (Fortune; Saunt, 2000).

Interestingly, apart from the common C_40_ carotenoids, in some *Citrus* species such as mandarins, oranges, and some mandarin hybrids, the presence of specific C_30_ apocarotenoid pigments has been reported (Fig. 1; Curl, 1965; Pfander *et al.*, 1980; Gross, 1987; Agócs *et al.*, 2007). The accumulation of C_30_ apocarotenoids is almost a unique feature of the genus *Citrus* and is rarely found in other plants or living organisms (Gross, 1987; Murillo *et al.*, 2012). C_30_ apocarotenoids appear to accumulate exclusively in the peel of mature fruits, since they have not been described in vegetative tissues and rarely and at low levels in the pulp (Gross, 1987; Oberholster *et al.*, 2001; Agócs *et al.*, 2007; Carmona *et al.*, 2012). The most abundant C_30_ apocarotenoid in *Citrus* is β-citraurin (3-hydroxy-β-apo-8′-carotenal; Fig. 1). This apocarotenoid was first identified by Zechmeister and Tucson (1936) and can be a major carotenoid accounting for up to 30% of total carotenoid content in the peel of some highly pigmented mandarin and mandarin hybrids (Farin *et al.*, 1983; Gross, 1987; Agócs *et al.*, 2007). Other C_30_ apocarotenoids are β-apo-8′-carotenal (Fig. 1), that has been isolated from the peel of some cultivars of mandarins and oranges (Winterstein *et al.*, 1960), and, of lower quantitative relevance, β-citraurinene (8′-apo-β-carotene-3-ol), β-citraurol (8′-apo-β-carotene-3,8′-ol), or β-citraurin epoxide (3-hydroxy-5,6-epoxy-5,6-dihydro-β-apo-8′-carotenal), which have also been reported in specific *Citrus* cultivars (Leuenberger and Stewart, 1976; Leuenberger *et al.*, 1976; Molnar and Szabolcs, 1980). β-Citraurin and, to a lower extent, β-apo-8′-carotenal, have been reported to be primary pigments in *Citrus* since they provide a bright reddish tint which contributes significantly to the characteristic deep orange-red coloration of the peel in some *Citrus* fruit (Fig. 1; Curl, 1965; Stewart and Wheaton, 1972; Gross, 1981; Farin *et al.*, 1983; Oberhoslter *et al*., 2001; Rios *et al.*, 2010; Carmona *et al.*, 2012).

The biosynthetic pathway leading to C_30_ apocarotenoids currently remains unknown, although biochemical data suggest that the β,β-xanthophyll β-cryptoxanthin is the most likely precursor since a direct substrate–product relationship has been described (Farin *et al.*, 1983). However, other carotenoids such as β-carotene or the xanthophyll zeaxanthin have also been proposed as potential precursors of β-apo-8′-carotenal or β-citraurin, respectively (Yokoyama and White, 1966; Rios *et al.*, 2010; Fig. 1). Based on the chemical structure, the hypothesized reaction to form β-citraurin or β-apo-8′-carotenal should be an asymmetric oxidative cleavage at the 7′,8′ double bond in the conjugated backbone of the parent carotenoid (Fig. 1).

The cleavage reactions of carotenoids are generally catalysed by a family of non-haem iron enzymes, so-called carotenoid cleavage oxygenases or dioxygenases (CCOs/CCDs), which are represented in all taxa (Bouvier *et al.*, 2005; Moise *et al.*, 2005). In plants, CCDs are generically grouped into five subfamilies according to the cleavage position and/or the substrate preference: NCEDs (nine-*cis* epoxy-carotenoid dioxygenases), CCD1s, CCD4s, CCD7s, and CCD8s (reviewed in Auldridge *et al*., 2006; Kloer and Schulz, 2006; Walter and Strack, 2011). The NCED group was the first characterized and they selectively act at the 11,12 bond of 9-*Z*-epoxycarotenoids to form xanthoxin (C_15_), which is the precursor of ABA (Schwartz *et al.*, 1997; Tan *et al.*, 1997). The CCD-type CCD7s and CCD8s act sequentially in the pathway leading to strigolactones (Ruyter-Spira *et al.*, 2013); first, CCD7 cleaves 9-*Z*-β-carotene eccentrically at the 9′,10′ position to generate a 9-*Z*-configured C_27_ intermediate (9-*Z*-β-apo-10′-carotenal), and β-ionone (C_13_). In the next step, CCD8 converts, in a presumed combination of different types of reactions, 9-*Z*-β-apo-10′-carotenal into carlactone, a C_19_ compound containing three oxygens and resembling strigolactones in function and structure (Alder *et al.*, 2012). Interestingly, CCD8 catalyses the typical cleavage reaction known from other CCDs when incubated with *E*-β-apo-10′-carotenal, yielding the C_18_ ketone β-apo-13-carotenone (Alder *et al.*, 2008). Enzymes of the CCD1 type are not predicted to be localized within the plastids. *In vitro*, CCD1 enzymes cleave a wide spectrum of cyclic and linear carotenoids and apocarotenoids at different symmetric positions (5,6/5′,6′;78/7′,8′; and 9,10/9′,10′) (Vogel *et al.*, 2008; Ilg *et al.*, 2009). However, it has been suggested that *in vivo* the CCD1 enzymes do not cleave intact carotenoids but may act coordinately with other CCDs, for example CCD7, to convert their apocarotenoid products further, for example C_27_ apocarotenoids (Ilg *et al*., 2009, 2010; Floss and Walter, 2009). The CCD4-type subfamily are localized in the plastid, and some members symmetrically cleave cyclic non-hydroxylated carotenoids at the 9,10(9′,10′) positions, while others may act preferentially on apocarotenoids at the 9,10 bond to produce C_13_ volatiles (Ohmiya *et al.*, 2006; Rubio *et al.*, 2008; Huang *et al*., 2009). A CCD-type enzyme from saffron, ZCD, has been reported to cleave zeaxanthin symmetrically at the 7,8(7′,8′) positions (Bouvier *et al.*, 2003). However, ZCD is a truncated version of the saffron CCD4a, which *in vitro* cleaves β-carotene at 9,10(9′,10′), and not zeaxanthin, like other CCD4-type enzymes (Rubio *et al.*, 2008). Therefore, to date no enzyme with an asymmetric 7,8 or 7′,8′ cleavage activity has been identified.

To date, in the genus *Citrus*, five *CCD* genes have been identified: two *NCED* genes that specifically cleave 9-*Z*-epoxycarotenoids and are involved in the biosynthesis of the phytohormone ABA (Kato *et al.*, 2006; Rodrigo *et al.*, 2006; Agustí *et al.*, 2007); one CCD1 type that fragments xanthophylls *in vitro* at the 9,10(9′,10′) position (Kato *et al.*, 2006); and two additional *CCD4-like* genes, *CCD4a* and *CCD4b*, whose activity has not been assessed (Agustí *et al.*, 2007). Therefore, despite the relevance of C_30_ apocarotenoids for the pigmentation of *Citrus* fruit, the gene/enzyme involved in their biosynthesis has not yet been identified. Here, the carotenoid cleavage enzyme responsible for the formation of *Citrus*-specific C_30_ apocarotenoids, which provide the characteristic and distinctive deep orange coloration in oranges and mandarin fruits, is reported.

## Materials and methods

### Plant material and fruit treatments

Fruits of Washington Navel sweet orange (*C. sinensis* L. Osbeck), Clemenules mandarin (*C. clementina*), and the hybrid Fortune mandarin (*C. clementina*×*C. reticulata*) were randomly harvested from adult trees cultivated at the Citrus Germplasm Bank (Instituto Valenciano de Investigaciones Agrarias, Moncada, Valencia, Spain). Fruits were harvested from immature-green to fully ripe stages in order to cover the developmental and ripening period. The fruit peel tissues (only the outer coloured part of the fruit peel) and the pulp were separated with a scalpel and collected. Adult leaves, young stems, roots, and petals (at anthesis) were collected from orange trees or seedlings as previously described (Alquézar *et al.*, 2009).

Ethylene degreening experiments were carried out on Navel sweet orange and Clemenules mandarin fruits. Fruits were harvested in October when they were showing a light green external colour, indicating that natural degreening was already initiated, and were subsequently incubated for 3 d in the dark in either an ethylene-free atmosphere (control fruit) or in an atmosphere with ethylene (10 µl l^–1^) in sealed 25 litre tanks at 20 ºC and 85–90% relative humidity. To avoid excess respiratory CO_2_, Ca(OH)_2_ powder was introduced into the tanks and fruit were ventilated every day. Peel was excised from the whole fruit and processed as described above at the onset of the experiment and after 3 d.

To investigate the effect of heat on apocarotenoid content and *CCD* gene expression, fruits of Navelina oranges (*C. sinensis*) were incubated at 37 ºC in the dark for 3 d. To maintain a high relative humidity during that period, a stream of humidified air was allowed to flow continuously through the container. Control fruits were incubated at 20 ºC under the same conditions. After 3 d of heat treatment, fruits were exposed to a continuous ethylene (10 µl l^–1^) treatment for 4 d at 20 ºC, as described above.

All plant material was frozen in liquid nitrogen immediately after harvest or after the different treatments, ground to a fine powder, and stored at –80 ºC until analysis.

### Analysis of carotenoids from Citrus tissues

Carotenoids from *Citrus* tissues were extracted as previously described by Rodrigo *et al.* (2003) and Alquézar *et al*. (2008*b*). Briefly, frozen ground material was extracted with a mixture of MeOH and 50mM TRIS-HCl buffer (pH 7.5) containing 1M NaCl and partitioned against chloroform until plant material was colourless. Pooled organic phases were dried under vacuum and saponified overnight using a KOH methanolic solution. The carotenoids were subsequently re-extracted with diethyl ether. Then, the extracts were reduced to dryness by rotary evaporation or nitrogen flow and kept under a nitrogen atmosphere at –20 ºC until high performance liquid chromatography (HPLC) analysis. Immediately prior to the injection in the HPLC system, carotenoid extracts were dissolved in a chloroform:MeOH:acetone (5:3:2) solution. Chromatography was carried out with a Waters liquid chromatography system equipped with a 600E pump and 996 photodiode array detector, and data were analysed with Empower software (Waters). Carotenoid pigments were separated by HPLC using a C_30_ carote column (250×4.6mm, 5 µm) coupled to a C_30_ guard column (20×4.0mm, 5 µm) (YMC Europe GMBH, Germany) with ternary gradient elution of MeOH, water, and *tert*-butyl methyl ether (TBME) (Rouseff *et al.*, 1996; Rodrigo *et al.*, 2003). The photodiode array detector was set to scan from 250nm to 540nm, and for each elution a Maxplot chromatogram, which plots each carotenoid peak at its corresponding maximum absorbance wavelength, was obtained.

Carotenoids were identified by their retention time, absorption, and fine spectra (Rouseff *et al.*, 1996; Britton, 1998*b*; Rodrigo *et al*., 2003, 2004). The carotenoid peaks were integrated at their individual maximal wavelength and their content was calculated using calibration curves of β-apo-8′-carotenal and β-citraurin (a gift from Hoffmann-LaRoche); β-carotene (Sigma) for α- and β-carotene; β-cryptoxanthin (Extrasynthese); lutein (Sigma) for lutein and violaxanthin isomers; and zeaxanthin (Extrasynthese) for zeaxanthin and antheraxanthin. Standards of phytoene and phytofluene for quantification were obtained from peel extracts of orange fruit as described in Alquézar *et al*. (2008*b*). The detection limit was estimated to be between 7ng and 15ng, depending on the carotenoid, which corresponded to ~30–60ng g^–1^ fresh weight (FW).

All operations were carried out on ice under dim light to prevent photodegradation, isomerizations, and structural changes of the carotenoids. Each sample was extracted at least in triplicate.

### Gene expression by quantitative reverse transcription–PCR (RT–PCR)

Total RNA was extracted from frozen *Citrus* tissues as described previously (Alquézar *et al.*, 2009) and subsequently treated with DNase I (DNA free, DNase treatment & removal, Ambion). The amount of RNA was measured by spectrophotometric analysis (Nanodrop) and its quality was verified by agarose gel electrophoresis and ethidium bromide staining. The absence of DNA contamination was checked by performing a no-reverse transcription assay which consisted of a PCR with each RNA sample using the *Citrus actin* primers (F-5′-TTAACCCCAAGGCCAACAGA-3′; R-5′-TCCCTCATAGATTGGTACAGTATGAGAC-3′). No amplified products were detected, which confirmed the purity of the RNA extracts. The transcripts present in 5 µg of total RNA were reverse-transcribed using the SuperScript III Reverse Transcriptase (Invitrogen) in a total volume of 20 µl. A 1 µl aliquot of a 10 times diluted first-strand cDNA was used for each amplification reaction.

Gene expression studies were performed following the MIQE guidelines (Bustin *et al.*, 2009). Quantitative real-time PCR was carried out on a LightCycler 480 instrument (Roche), using the LightCycler 480 SYBRGreen I Master kit (Roche). Reaction mix and conditions followed the manufacturer’s instructions, with some modifications. The PCR mix contained 1 µl of diluted cDNA, 5 µl of SYBR Green I Master Mix, 1 µl of 3 µM primer F, and 1 µl of 3 µM primer R, the final volume being 10 µl. The primers (PSF purified, Isogen) used for the amplification of each gene were: CCD4a-F-5′-GGACGGACCTTGTCCACGCG-3′, CCD4a-R-5′-GCAATCCC GACGCTCGTCGCC-3′, CCD4b1-F-5′-CAGCAAGAAATTTG GAGTTG-3′, CCD4b1-R-5′-CGTAAAATCTTCTTGAGAC-3′, CCD4b2-F-5′-CAGCAAGAAATTTGGAGTTG-3′, CCD4b2-R-5′- AGCAGCATCCAGATGATCTT-3′, CCD4c-F-5′-GGAGTAGTTTCAAGGCATCC-3′, and CCD4c-R-5′-GTAGCCACAGTGCACTCCCG-3′. The cycling protocol, for all genes, consisted of 10min at 95 °C for pre-incubation, then 40 cycles of 10 s at 95 °C for denaturation, 10 s at 60 °C for annealing, and 10 s at 72 °C for extension. Fluorescent intensity data were acquired during the extension time with the LightCycler 480 Software release 1.5.0, version 1.5.0.39 (Roche) and were transformed into mRNA levels by using specific standard curves for all analysed genes (CP values ranging between 22 and 29). Gene expression was not considered in samples showing a CP value >32. The specificity of the PCR was assessed by the presence of a single peak in the dissociation curve performed after the amplification steps followed by the sequencing of the amplicon. Three potential housekeeping genes were tested in this experiment based on previously published primer sequences of *Citrus* genes: *actin* (*ACT*), *β-tubulin* (*β-TUB*) (Romero *et al.*, 2012), and *elongation factor 1* (*EF1*) (Distefano *et al.*, 2009). To test the stability of these genes in these particular samples, the BestKeeper software was used (Pfaffl *et al.*, 2004). In this analysis, the most stably expressed genes are those having the lowest SD [± CP], and any studied gene with an SD >1 can be considered inconsistent. The *ACT* gene which had the lowest SD [± CP] value (0.38) was the best housekeeping gene for the present analysis and this is the reason for the normalization against it (Pfaffl *et al.*, 2004).

The expression levels relative to values of a reference sample were calculated using the Relative Expression Software Tool (REST, http://rest.gene-quantification.info; Pfaffl *et al.*, 2002).

### Cloning Citrus CCD4b1 for in vitro assays

Total RNA from coloured Clementine mandarin peel was used to prepare cDNA as indicated in the previous section. The cDNA was then used to amplify the open reading frame (ORF) of *CCD4b1* without the first 41 amino acids which are predicted to be the transit peptide for plastid localization. The *CCD4b1* amplification was performed using the primers MJ145 (F-5′-GTGGCACCCATACAATCTTTAATGGGAACAAATTC-3′) and MJ146 (F-5′-TCATAAACTGTTTTGGTTTGAGTGATTCTC-3′) and a high-fidelity polymerase (KAPA HiFi DNA Polymerase, KapaBiosystems). The resulting amplicon was gel purified using the UltraClean DNA purification kit (MO BIO Laboratories). The purified *CCD4b1* was subsequently amplified using the primers MJ301 (F-5′-CGCCCTTGGCGAATTCGCACCCATACAATCTTTAATGGG-3′) and MJ300 (F-5′-TACCCTCGAGGAATTCCGGGAATTCGATTTCATAAACTC-3′) and a high-fidelity polymerase (KAPA HiFi DNA Polymerase, KapaBiosystems) in order to clone this gene in-frame with the thioredoxin gene into the pBAD-Thio vector (Invitrogen) by recombination using the In-Fusion^®^ HD Cloning Plus CE kit (Clontech). The resulting expression plasmid, namely pThio-*CCD4b1*, was sequenced to confirm the correct assembly and lack of sequence errors. Then, pThio-*CCD4b1* was transformed into *Escherichia coli* BL21 (Tuner DE3, Novagen) cells harbouring pGro7, a plasmid encoding the groES–groEL–chaperone system under the control of an arabinose-inducible promoter (Nishihara *et al.*, 1998; TAKARA BIO INC.). Transformed cells were grown, induced, and prepared to obtain crude lysates according to Alder *et al.* (2008).

### In vitro assays

Apocarotenoids and β-cryptoxanthin were kindly provided by the BASF (Ludwigshafen, Germany), and β- and α-carotene were purchased from Sigma-Aldrich (Deisenhofen, Germany) and Carotenature (Lupsingen, Switzerland), respectively. Substrates were purified by thin-layer chromatography (Ruch *et al.*, 2005) and quantified spectrophotometrically at their individual λ_max_ using extinction coefficients as described in Davies (1976) or Barua and Olson (2000). *In vitro* assays were carried out with a substrate concentration of 40 µM, according to Scherzinger and Al-Babili (2008) and using 50 µl of crude lysate [50mM sodium phosphate pH 8.0, 300mM NaCl, 1mg ml^–1^ lysozyme, 1mM dithiothreitol (DTT), 0.1% Triton X-100] obtained from overexpressing cells in a total reaction volume of 200 µl. Substrates were mixed with 20 µl of 2% Triton X-100 in EtOH (final assay concentration 0.2%, v/v), dried in a vacuum centrifuge, and finally resuspended in 50 µl of H_2_O. The obtained micelles were mixed with 100 µl of 2× incubation buffer consisting of 100mM HEPES pH 7.8, 1mM TCEP, 0.2mM FeSO_4_, and 1mg ml^–1^ catalase. A 50 µl aliquot of crude lysate was then added and assays were incubated at 28 °C under shaking (200rpm, ThermoMixer MKR 13, DITABIS, Germany) in complete darkness. Assays were stopped after either 3h (for lycopene) or 1h (for other substrates) by adding 2 vols of acetone, and lipophilic compounds were partitioned against 600 µl of petroleum ether/diethyl ether 1:4 (v/v), vacuum-dried, and dissolved in 40 µl of chloroform for HPLC analysis.

### HPLC and gas chromatography–mass spectrometry (GC-MS) analysis for CCD4b1 *in vitro* assays

HPLC analysis was performed with 5 µl of extracts on a Shimadzu UFLC XR equipped with an SPD-M20A PDA (Duisburg, Germany) and a C_30_ column (150×3mm, 5 µm) (YMC, Germany), using the solvent systems A, MeOH:TBME (1:1); and B, MeOH:TBME:H_2_O (30:1:10). The gradient was developed at a flow rate of 0.6ml min^–1^ from 100% B to 100% A in 20min, maintaining 100% A for 4min and followed by re-equilibration to initial conditions in 6min.

GC-MS analysis was performed on a Thermo Scientific Trace GC equipped with a DSQ II mass spectrometer and a thermodesorber UNITY2, from MARKES International. The GC column was a Zebron ZB-5 (15 m×0.25mm, 0.25 µm film thickness).

Thermodesorption settings were as follows: tube desorption at 280 °C for 5min with a trap flow of 20ml min^–1^. Trap desorption was performed from –10 °C to 360 °C (100 °C s^–1^) with final conditions maintained for 10min and a trap flow of 20ml min^–1^. The GC gradient started with an initial temperature of 60 °C held for 2min and followed by a ramp to 320 °C within 25min, with final conditions maintained for 5min.

## Results

### Identification of the CCD4-like gene family in Citrus sp.

In order to identify the enzyme(s) responsible for the biosynthesis of *Citrus*-specific C_30_ apocarotenoids, a tBLASTN search in the Sweet Orange Genome Project (2010) (http://www.phytozome.net/), the Haploid Clementine Genome International Citrus Genome Consortium (2011) (http://int-citrusgenomics.org/, http://www.phytozome.net/clementine), and the ‘double haploid sweet orange genome project’ (http://citrus.hzau.edu.cn/orange/) (Xu *et al.*, 2013) using the sequence of the zeaxanthin cleavage dioxygenase (ZCD; accession no. Q84K96) from *Crocus sativus* was carried out. This protein was selected since it has been reported to cleave cyclic carotenoids at the 7,8(7′,8′) positions (Bouvier *et al.*, 2003), a reaction similar to that proposed for *Citrus* C_30_ apocarotenoid biosynthesis. The best hits (58% identity at the protein level) were obtained for the gene Ciclev0031003m.g of *C. clementina* and 1.1g044599m/Cs7g14820.1 of *C. sinensis* (Table 1), which were identical to the *CCD4a* (ABC26012) gene previously isolated from a *C. clementina* cDNA library (Agustí *et al.*, 2007). A second hit (50% identity to ZCD) was identified in *C. clementina* (Ciclev10028113m.g) and *C. sinensis* (1.1g040986m/Cs8g14150) genomes (Table 1) that were 99.5% identical to the previously described *CCD4b* gene (ABC26011) from *C. clementina* (Agustí *et al.*, 2007). Three additional genes related to ZCD were also found in the *Citrus* genomes: a gene highly similar to *CCD4b* (88% identity at the protein level) and therefore named *CCD4b2* (Ciclev0030384m.g and 1.1g046348m/Cs8g141808.1) (Table 1), and two more *CCD4-like* genes that were designated as *CCD4c* and *CCD4d* (Table 1). The five genes identified in the genomes of clementine and sweet orange were grouped in the *CCD4-like* family, and, following the previous nomenclature, were named *CCD4a*, *CCD4b1* (formerly *CCD4b*), *CCD4b2*, *CCD4c*, and *CCD4d* (Table 1). The proteins CCD4a, CCD4b1, and CCD4c were almost identical (98–100% identity) between the two genotypes of mandarin and orange, while CCD4b2 and CCD4d of orange were shorter than those of mandarin, with CCD4d showing a truncation of ~100 amino acids in its C-terminus.

**Table 1.** Genomic and structural characteristics of CCD4-like gene family in Citrus

Gene name	Genomic code, *Citrus clementina* ^*a*^	Genomic code, *Citrus sinensis* (SOGP^*b*^/DHSOG^*c*^)	Predicted introns (C.c^*a*^/C.s^*bc*^)	Predicted protein length (amino acids) (*C. clementina/C. sinensis*)	Chloroplast transit peptide^*d*^	EST *Citrus* libraries^e^
*CCD4a*	Ciclev10031003m.g	orange1.1g044599m/ Cs7g14820.1	0	603/603	Yes	Yes
*CCD4b1*	Ciclev10028113m.g	orange1.1g040986m/ Cs8g14150	0	563/563	Yes	Yes
*CCD4b2*	Ciclev10030384m.g	orange1.1g046348/ Cs8g14180.1	0	557/358^*b*^ or 418^*c*^	Yes	No
*CCD4c*	Ciclev1001335m.g	orange1.1044992m/ Cs6g19500.1	0	597/597	Yes	Yes
*CCD4d*	Ciclev10013726m.g	orange1.1g0339955m/ Cs6g19550	2/4	412/420	Yes	No

^*a*^ Haploid Clementine Genome International Citrus Genome Consortium (2011).

^*b*^ Sweet Orange Genome Project (2010).

^*c*^ Double haploid sweet orange genome (Xu *et al.*, 2013).

^*d*^ ChloroP 1.1. Prediction Server.

^*e*^ HarvEST database 1.32, Assembly C52.

The organization of the sweet orange haploid genome in pseudochromosomes allowed the positioning of the different *CCD4-like* genes. Thus, *CCD4a* was located on chromosome 7, *CCD4b1* and *CCD4b2* were both on chromosome 8 separated by 45kb, and *CCD4c* and *CCD4d* were on chromosome 6 separated by 20kb. Interestingly, no introns were identified in any of the *CCD4* genes except for *CCD4d*, which is predicted to have two and four introns in *C. clementine* and *C. sinensis*, respectively. Consistent with the putative carotenoid cleavage activity, all the identified proteins contained at the N-terminus a plastid transit peptide consisting of 30–45 amino acids (Table 1).

**Fig. 2.**
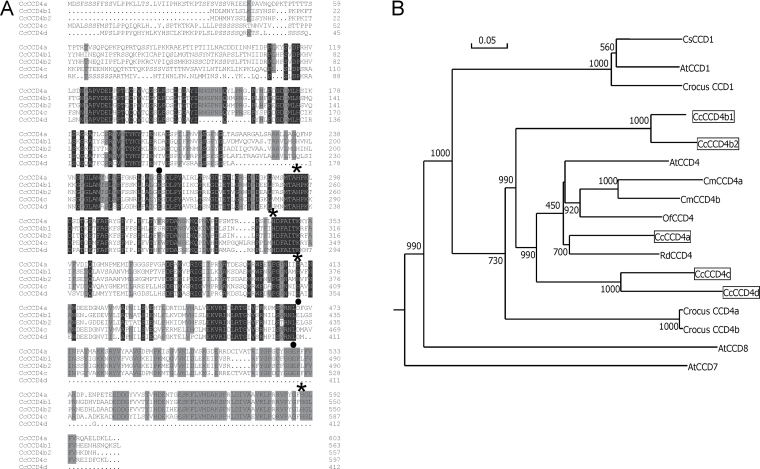
Alignment of *Citrus clementina* CCD4-like proteins (A) and phylogenetic tree of *Citrus* CCD4-like and other plant CCDs (B). (A) The alignment of *Citrus* CCD4-like proteins was created using the CLUSTAL W program (Thompson *et al.*, 1994). Numbers on the right denote the number of amino acid residue. Residues identical for all the sequences in a given position are in white text on a black background, and 75–100% homologous residues are presented on a grey background. The asterisks indicate the histidine residues involved in the coordination of the catalytic Fe^2+^ and black dots indicate aspartate or glutamate residues which are predicted to be fixing the iron atom. (B) The phylogenetic tree was generated based on the alignment of deduced amino acid sequences of *C. clementina* CCD4-like proteins and other plant CCDs. The tree was constructed on the basis of the Neighbor–Joining method (Saitou and Nei, 1987). The bootstrap values on the nodes indicate the number of times that each group occurred with 1000 replicates. The sequences used to generate the phylogenetic tree and their accession numbers are as follows: *Citrus sinensis* CsCCD1 (accession no. BAE92958); *Arabidopsis thaliana* AtCCD1 (accession no. AT3G63520), AtCCD4 (accession no. O49675), AtCCD7 (accession no. NP_195007), and AtCCD8 (accession no. NM_130064); *Crocus sativus* CCD1 (accession no. CAC_79592), CCD4a (accession no. EU523662), and CCD4b (accession no. EU523663); *Citrus clementina* CcCCD4a (accession no. Ciclev10031003m), CcCCD4b1 (accession no. Ciclev10028113m), CcCCD4b2 (accession no. Ciclev10030384m), CcCCD4c (accession no. Ciclev1001335m), and CcCCD4d (accession no. Ciclev10013726m); *Chrysanthemum morifolium* CmCCD4a (accession no.AB247148) and CmCCD4b (accession no. AB247160); *Osmanthus fragans* OfCCD4 (accession no. EU334434); and *Rosa damascena* RdCCD4 (accession no. EU334433).

The relationship between the predicted CCD4-like proteins from *Citrus* and other plant CCDs including several CCD4, CCD1, CCD7, and CCD8 proteins, was analysed by sequence comparison and by the generation of a phylogenetic tree (Fig. 2). All *Citrus* CCD4s fitted in the CCD4 group but were located in two different clusters: CCD4a, CCD4c, and CCD4d were grouped with *Osmanthus*, *Chrysanthemum*, *Arabidopsis*, and *Rosa* CCD4 proteins that cleave cyclic carotenoids or apocarotenoids at the 9,10 and/or 9′,10′ positions to release the C_13_ β-ionone (Huang *et al*., 2009). CCD4b1 and CCD4b2 were located in a separate branch and more distantly related to other CCD4 proteins (Fig. 2). Interestingly, none of the *Citrus* CCD4s was clustered with saffron CCD4a and CCD4b, which are longer versions of ZCD, and cleave β-carotene symmetrically at the 9,10(9′,10′) double bonds (Rubio *et al.*, 2008).

The comparison of the full protein sequences of *Citrus* CCD4-like members revealed that the highest variability among them was located in the N-terminus, the region where the transit plastid peptide is predicted (Fig. 2). CCD4b1 and CCD4b2 were the most closely related sequences (88% identity), and the least similar to CCD4a and CCD4c (48% and 45% identity, respectively) (Supplementary Table S1 available at *JXB* online). It is noteworthy that CCD4a and CCD4c shared 60% identity (Supplementary Table S1), a percentage similar to that observed among other CCD4s from different plant species (Ahrazem *et al.*, 2010). As expected, CCD4d was the protein with the lowest homology with the other *Citrus* CCDs (30–49% of identity) since it lacks ~100 amino acids at the C-terminus (Fig. 2). Several conserved motifs among CCDs were found in *Citrus* CCD4a, CCD4b1, and CCD4c, such as the four histidines coordinating the Fe^2+^ cofactor required for activity, or the aspartate or glutamate residues fixing the positions of histidines (Fig. 2) (Schwartz *et al.*, 1997; Huang *et al*., 2009; Messing *et al.*, 2010). In CCD4d from *C. clementina* and *C. sinensis*, and also in CCD4b2 from *C. sinensis*, important functional residues for CCD activity were missing, including one of the histidines that coordinates the cofactor Fe^2+^.

In order to investigate the representation of the *CCD4-like* genes in *Citrus* transcriptomes, data mining of a specific *Citrus* expressed sequence tag (EST) database (http://harvest.ucr.edu/, software HarvEST 1.32, assembly C52) containing a collection of 469.618 ESTs (40.438 contigs) from 141 *Citrus* cDNA libraries was carried out. The *in silico* analysis revealed the presence of ESTs corresponding to *CCD4a*, *CCD4b1*, and *CCD4c*, while, in contrast, ESTs for *CCD4b2* and *CCD4d* were absent. Twenty-six ESTs matching with *CCD4b1* were found, and it was remarkable that all ESTs were identified exclusively in libraries obtained from tissue of fruit peel or a mixed library containing different fruit tissues. A total of 12 ESTs corresponding to *CCD4a* were identified in seven libraries prepared from leaf tissues, whole plant, or a mixture of tissues. Only four ESTs for *CCD4c*, which were obtained from a single library prepared from a mixture of several tissues, were found. These results suggest that only *CCD4a*, *CCD4b1*, and *CCD4c* are actually expressed, and that probably *CCD4b2* and *CCD4d*, which are located on the same chromosomes as *CCD4b1* and *CCD4c*, respectively, might be pseudogenes.

### Expression analysis of CCD4-like genes in different reproductive and vegetative Citrus tissues

**Fig. 3.**
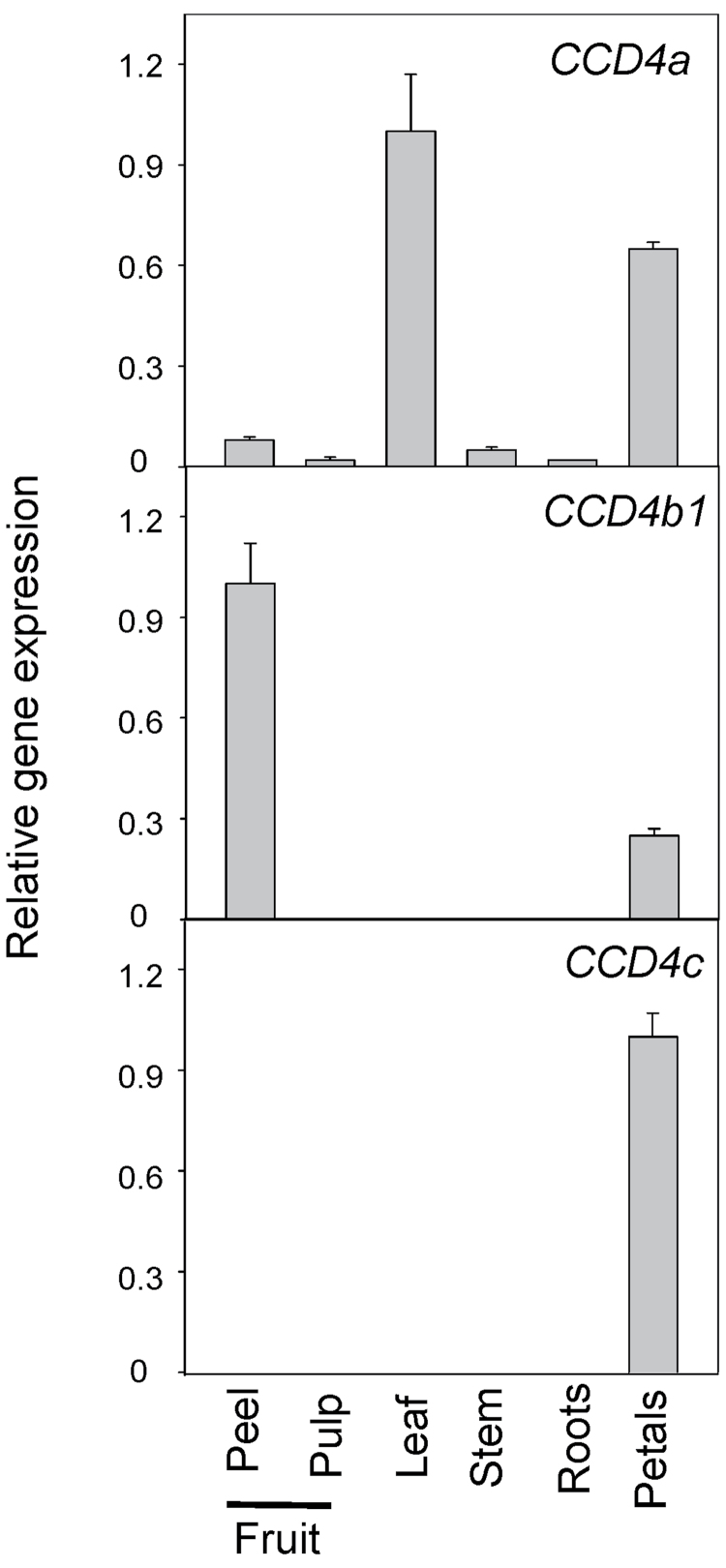
Expression of *CCD4a*, *CCD4b1*, and *CCD4c* genes in different vegetative and reproductive tissues of Navel sweet orange (*Citrus sinensis*). For each gene, the expression values are relative to the sample with the maximum expression level which was set to 1. The data are means ±SD of three experimental replicates.

Accumulation of *Citrus* C_30_ apocarotenoids is restricted to the fruits, while other reproductive or vegetative *Citrus* tissues do not show detectable amounts of these pigments. Within the fruits, C_30_ apocarotenoids are mainly present in the peel, while pulp contains only low amounts of these compounds or lacks them (Curl *et al*., 1965; Gross, 1987; Agócs *et al.*, 2007). In order to identify the gene(s) encoding the enzyme which catalyses the cleavage reaction leading to C_30_ apocarotenoids, apocarotenoid analysis was first performed and the transcript levels of *CCD4a*, *CCD4b1*, *CCD4b2*, and *CCD4c* were determined in different reproductive and vegetative tissues of sweet orange, to check for spatial correlation with apocarotenoid accumulation. The *Citrus CCD4d* was excluded from the analysis since the predicted protein is truncated at the C-terminus, lacking, among other putative functional motifs, a histidine essential for the cleavage activity (Fig. 2). As expected, the only C_30_ apocarotenoid identified was β-citraurin, and its presence was exclusively restricted to fruit peel. The *CCD4b1* and *CCD4c* genes showed a tissue-specific pattern of expression: *CCD4c* was only detected in petals, a white tissue devoid of coloured carotenoids, while *CCD4b1* was predominantly expressed in fruit peel and at much lower levels in petals (Fig. 3). The expression of *CCD4a* was ubiquitous in all tissues analysed, although, as reported for other plant CCD4-type enzymes (Huang *et al*., 2009; Campbell *et al.*, 2010), the accumulation of the transcript was substantially higher in leaves and petals (Fig. 3). *CCD4b2* expression was not detected in any of the tissues/organs analysed, corroborating the results of the *in silico* analysis where no ESTs corresponding to *CCD4b2* were identified. Taken together, the expression profile of the four *CCD4-like* genes from *Citrus* suggests that *CCD4b1* is the most promising candidate for the biosynthesis of C_30_ apocarotenoids since this is the only gene which shows the highest expression in fruit peel coinciding with the observation that β-citraurin is restricted to this tissue. Moreover, the absence of *CCD4b2* and *CCD4c*, or extremely low expression of *CCD4a* genes in fruit peel tissue may indicate that they are probably involved in other carotenoid cleavage reactions unrelated to the biosynthesis of C_30_ apocarotenoids.

### Expression of CCD4b1 in peel and pulp of Citrus fruit and the relationship with the accumulation of C_30_ apocarotenoids

#### Fruit development and ripening

**Fig. 4.**
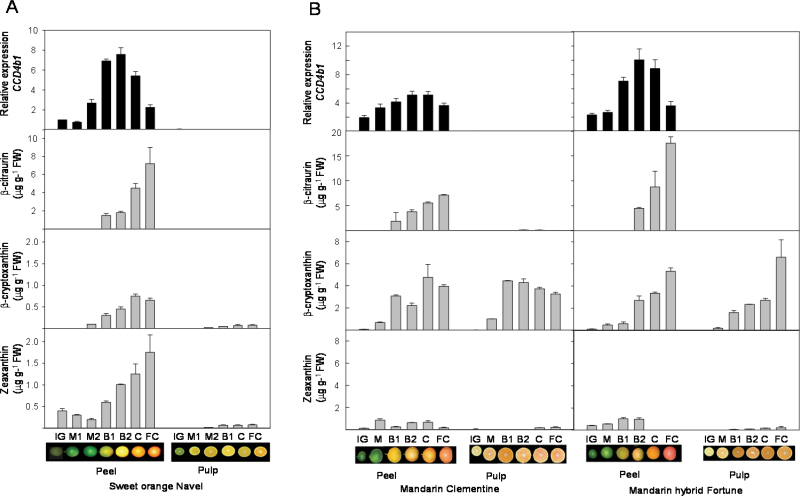
Transcript levels of the *CCD4b1* gene and the accumulation of the C_30_ apocarotenoid β-citraurin, in the peel and pulp of Navel sweet orange (A), Clementine mandarin, and the hybrid Fortune mandarin (B) during fruit development and ripening. Changes in the concentration of the putative precursors of β-citraurin, β-cryptoxanthin, and zeaxanthin, are also shown, and for comparative purposes the same scale for both xanthophylls is used. The stages of fruit development and ripening are: IG, immature green; M1 and M2, mature green; B1 and B2, breaker; C, coloured; and FC, fully coloured fruit, as described in Alquézar *et al*. (2008*b*). Expression values are relative to transcript levels obtained in the peel of sweet orange at the IG stage which was arbitrarily set to 1. Data of carotenoid content and transcripts accumulation are means ±SD of three replicates.

To explore in depth the putative involvement of *CCD4b1* in the biosynthesis of fruit-specific C_30_ apocarotenoids, the expression of *CCD4b1* was analysed in peel and pulp tissues during fruit development and ripening, and compared with the content of C_30_ apocarotenoids. Moreover, in order to corroborate the involvement of C_30_ apocarotenoids in the intensity of pigmentation in the peel of *Citrus* fruits, the content of apocarotenoids and the expression of *CCD4b1* were analysed in fruits of three different genotypes selected by their marked differences in external fruit coloration: Navel sweet orange (*C. sinensis*), Clementine mandarin (*C. clementina*) both showing a moderate coloration, and the hybrid Fortune mandarin (a cross between the mandarins *Citrus clementina* cv. Fino*×Citrus reticulata* cv. Dancy) that is well recognized by its intense reddish-orange fruit peel (Saunt, 2000; http://www.citrusvariety.ucr.edu/citrus/fortune.html) (Fig. 4).

The content of β-citraurin was determined in peel and pulp during development and ripening of the three genotypes (Fig. 4). No β-citraurin was detected in the peel of green fruits and in pulp samples at any developmental stage. In the peel of the three genotypes, β-citraurin was the main C_30_ apocarotenoid identified and its concentration increased from breaker to fully ripe stage, showing the maximum level in the peel of fully coloured fruits (Fig. 4). In fully coloured fruit, the highest concentration of β-citraurin was detected in peel of the hybrid Fortune, which was more than twice (17 µg g^–1^ FW) than in sweet orange and Clementine mandarin (~7 µg g^–1^ FW) (Fig. 4). Moreover, it is interesting that the peel of Clementine mandarin fruits at breaker stage also contains traces of β-apo-8′-carotenal (<1 µg g^–1^ FW). With the aim of finding a possible substrate–product relationship, the amounts of β-cryptoxanthin and zeaxanthin, the two xantophylls postulated as β-citraurin precursors (Gross, 1987), were also determined. In the three genotypes, the content of β-cryptoxanthin was low in green tissues and increased substantially in both peel and pulp during ripening. However, the concentration of β-cryptoxanthin in both mandarins was much higher (~10-fold in the peel and 100-fold in the pulp) than in sweet orange (Fig. 4). In addition, it was also observed that the β-cryptoxanthin concentration in the peel and the pulp of mandarins was similar, while in sweet orange this carotenoid occurred at a significantly higher level in the peel than in the pulp (Fig. 4). These results are in good agreement with the well-known phenomena that mandarin juice vesicles accumulate elevated concentrations of β-cryptoxanthin (Kim *et al.*, 2001; Kato *et al.*, 2004; Fanciullino *et al.*, 2006; Xu *et al.*, 2006). The content of zeaxanthin in the pulp of the three genotypes was very low (<0.2 µg g^–1^ FW) throughout the whole development and ripening period. The changes in zeaxanthin concentration in the peel differed substantially among the genotypes. In sweet orange, the zeaxanthin content was low in green fruits and increased progressively during ripening. In contrast, in Clementine mandarin peel, the zeaxanthin content fluctuated without a specific pattern, and in the hybrid Fortune the concentration of this xanthophyll slightly increased until breaker stage, but became undetectable in the peel of fully coloured fruits (Fig. 4).

Expression of *CCD4b1* exhibited a similar pattern during fruit development and ripening in the three genotypes. In the peel, the lowest transcript level was detected at the green stages, and a substantial increment, more evident in sweet orange and in the mandarin hybrid, was achieved at the breaker stage coinciding with transition from chloroplasts to chromoplasts (Fig. 4A). The highest level was then reached at late breaker stage or at the initiation of coloration, and then decreased progressively at the end of the ripening period (fully coloured fruit) (Fig. 4). The comparison of the relative expression level of *CCD4b1* between the peel of the different genotypes revealed that at breaker stage the mandarin hybrid and sweet orange reached the highest expression levels which were 2- and 1.5-fold higher, respectively, than in Clementine mandarin at the same developmental stage. Interestingly, no *CCD4b1* transcripts were detected in any of the pulp samples analysed of the three species, in agreement with the previous results (Fig. 3).

#### Effect of treatments that modify Citrus fruit colour

**Fig. 5.**
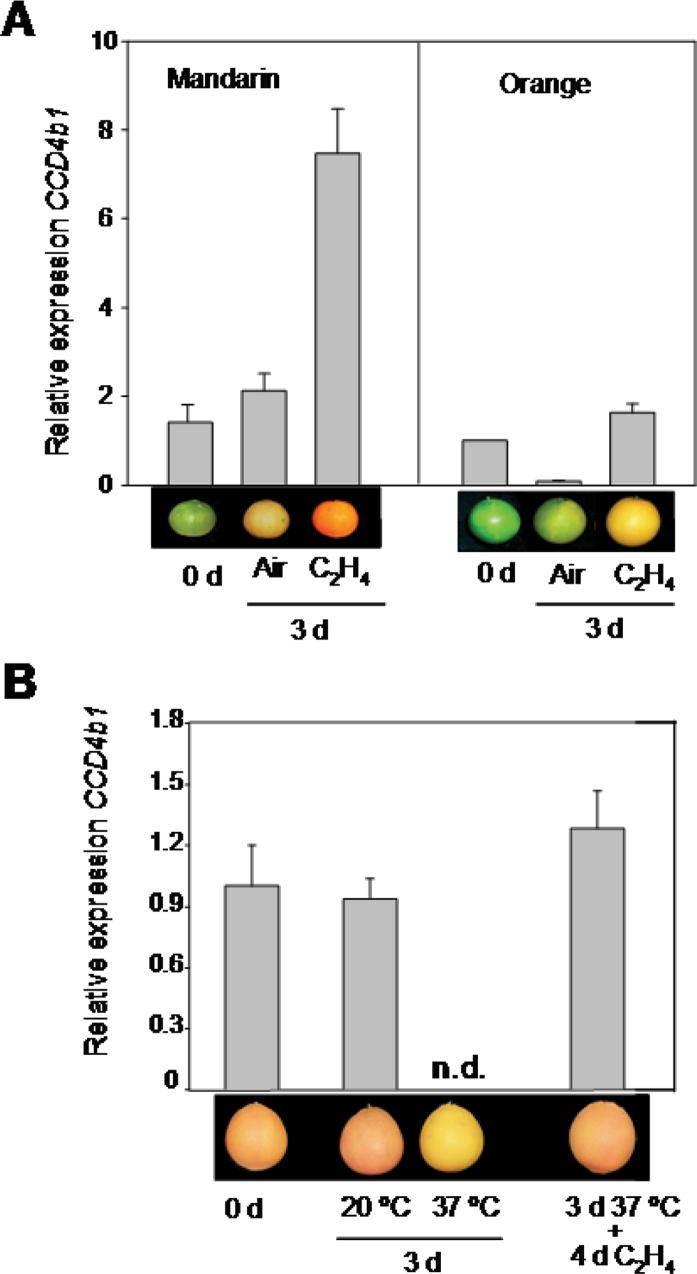
Effect of a continuous ethylene treatment (A) and heat treatment (37 ºC) followed by ethylene application (B) on the expression of the *CCD4b1* gene in peel of Navel oranges or Clementine mandarin. (A) Mature-green fruits (harvested in October) of Navel sweet orange and Clementine mandarin were exposed to air or to ethylene (10 µl l^–1^) at 20 ºC for up to 3 d. (B) Coloured Navel sweet oranges (harvested in November) were incubated at 20 ºC (control) or heat treated at 37 ºC and 90% relative humidity for 3 d. After heating at 37 ºC, fruits were exposed to an ethylene treatment (10 µl l^–1^) at 20 ºC for an additional 4 d. The images are representative of the external colour of the fruits at the beginning and the end of each treatment. Expression values are relative to transcript levels obtained in the peel of Navel sweet orange fruits at the onset of the experiments, which was arbitrarily set to 1. N.d. indicates not detected. Data of transcripts levels are means ±SD of three replicates. (This figure is available in colour at *JXB* online.)

Exogenous application of ethylene is a worldwide commercial post-harvest practice employed to promote peel degreening of *Citrus* fruits to enhance their external coloration (Stewart and Wheaton, 1972; Young and Otto, 1972; Eilati *et al.*, 1975; Wardowsky *et al.*, 2006). Ethylene reproduces and accelerates the changes in carotenoid composition in the peel which occur naturally during fruit ripening, including the increase in β-citraurin content (Stewart and Wheaton, 1972; Gross, 1981; Rodrigo and Zacarías, 2007). Therefore, experiments were carried out to investigate whether the ethylene-induced peel coloration and β-citraurin enhancement is accompanied by an increase in *CCD4b1* transcript level. For this purpose, mature-green fruits of Navel orange and Clementine mandarins were treated with ethylene (10 µl l^–1^) for 3 d or maintained in an air atmosphere (control), and the carotenoid composition and *CCD4b1* transcript levels in the fruit peel were determined. Continuous ethylene application accelerated peel coloration in both genotypes, which reached an intense orange colour in mandarin or a pale yellowish tint in orange (Fig. 5A). This treatment greatly affected the carotenoid composition in the peel of both genotypes (Table 2). Accumulation of β-citraurin occurred in both air- and ethylene-treated mandarin fruit, although in the latter the concentration was about three times higher. Concomitantly, a reduction in zeaxanthin content was also detected. A second C_30_ apocarotenoid, β-apo-8′-carotenal, was also present in the peel of mandarin fruits, but only at an early stage of the treatment (Table 2). In orange fruit, accumulation of β-citraurin was only detected in ethylene-treated fruit, in accordance with the effect on fruit colour (Table 2). Ethylene also induced a significant reduction in the concentration of chloroplast-type carotenoids, namely lutein, β- and α-carotene, all-*E*-violaxanthin, and neoxanthin, and an increase in phytoene. In sweet orange, ethylene also stimulated the accumulation of 9-*Z*-violaxanthin, zeaxanthin, and, to a lesser extent, β-cryptoxanthin (Table 2).

**Table 2.** Effect of ethylene (C_2_H_4_) treatment on carotenoid composition and C_30_ apocarotenoid content in the peel of sweet orange (cv. Navel) and mandarin (cv. Clementine) harvested at the onset of natural degreeningFruits were harvested in October with an initial green or light green colour for sweet orange and mandarin, respectively, and exposed to air or 10 µl l^–1^ ethylene at 20 ºC for 3 d.

Carotenoid (µg g^–1^ FW)	Sweet orange			Mandarin		
	0 d	3 d		0 d	3 d	
		Air	C_2_H_4_		Air	C_2_H_4_
C_40_						
Phytoene	0.61±0.10	0.82±0.05	3.21±0.52	1.04±0.08	1.74±0.20	5.57±0.85
β-Carotene	0.41±0.008	ND	ND	3.20±0.52	ND	ND
α-Carotene	Traces^*a*^	ND	ND	2.23±0.26	ND	ND
β-Cryptoxanthin	ND	ND	0.15±0.04	1.52±0.26	1.69±0.33	2.30±0.29
Zeaxanthin	Traces	0.54±0.18	0.94±0.11	0.75±0.09	0.46±0.07	0.25±0.04
*all-E*-Violaxanthin	4.15±0.70	3.27±0.61	1.57±0.25	28.17±0.79	17.30±0.81	11.70±0.59
9-*Z*-Violaxanthin	5.21±0.56	6.21±0.21	15.24±2.21	27.95±3.98	45.99±2.50	33.74±3.50
Neoxanthin	2.25±0.16	1.74±0.56	ND	6.04±2.36	2.41±0.09	ND
Lutein	6.21±0.62	1.21±0.32	0.40±0.06	21.32±4.17	ND	ND
C_30_						
β-Apo-8′-carotenal	ND	ND	ND	0.55±0.13	ND	ND
β-Citraurin	ND	ND	2.31±0.08	Traces	2.79±0.27	6.13±0.48

The values are means ±SD of at least three measurements.

ND, not detected.

^*a*^ Traces: carotenoid identified by retention time and spectrum characteristics but their peak area was too low for a reliable quantification.

The effect of ethylene on the *CCD4b1* transcript levels was different in the peel of both genotypes but consistent with β-citraurin accumulation. In mandarin, the level of *CCD4b1* mRNA was noticeably up-regulated upon ethylene treatment and to a lesser extent in air-treated fruits (4- and 3-fold, respectively) (Fig. 5A). In orange fruits, *CCD4b1* gene expression was only moderately stimulated by ethylene, but it was dramatically reduced in the peel of air-treated samples (Fig. 5A).

In order to find additional evidence for the correlation of the *CCD4b1* transcript level, β-citraurin accumulation, and coloration of *Citrus* fruits, a second treatment was selected, based on the detrimental effect of high pre- and post-harvest temperatures (>30 ºC) suppressing peel coloration (Young and Erickson, 1961; Stewart and Wheaton, 1972; Jahn *et al.*, 1973; Cohen, 1978; Plaza *et al.*, 2004). Therefore, coloured sweet orange fruit were exposed to a post-harvest heat treatment at 37 ºC for 3 d, and the carotenoid composition and expression of the *CCD4b1* gene were determined and compared with those of control fruits incubated at 20 ºC for the same period of time. Moreover, to explore the possibility that ethylene may reverse the detrimental effect of high temperatures on fruit colour, previously heated fruits were subsequently exposed to an ethylene treatment for 4 d. The heat treatment provoked a visible change in the external pigmentation of sweet orange, resulting in light yellow coloration (Fig. 5B). No significant effect was observed in fruit exposed to 20 ºC. The carotenoid composition in the peel of heated fruits was dramatically affected, and a decrease by 6- and 9-fold in β-citraurin, relative to the initial content or that of fruits exposed to 20 ºC, respectively, was detected (Table 3). It is noteworthy that heat treatment also promoted a 2-fold increment in β-cryptoxanthin, although the concentration in orange peel still remained at relatively low levels (<0.5 µg g^–1^ FW) (Table 3). The application of exogenous ethylene reversed the effect of heat on fruit coloration and stimulated external peel colour (Fig. 5B) that was associated with a recovery of the β-citraurin level (Table 3). This ethylene treatment also provoked a decline in β-cryptoxanthin content and a significant increment in early colourless carotenes (phytoene and phytofluene) (Table 3). The expression of the *CCD4b1* gene was dramatically abolished in the peel of fruits incubated for 3 d at 37 ºC, while it was not affected at 20 ºC (Fig. 5B). The exposure of heat-treated fruits to ethylene stimulated *CCD4b1* expression and recovered initial transcript levels (Fig. 5B). In coloured control fruits incubated at 20 ºC, ethylene only stimulated the content of upstream carotenes (phytoene and phytofluene) and did not alter *CCD4b1* gene expression (data not shown).

**Table 3.** Effect of heat treatment (37 ºC at 90% relative humidity) on the content of the main carotenoids and C_30_ apocarotenoid in the peel of sweet orange (cv. Navelina)Coloured fruits were harvested in November and stored at 20 ºC (control) or at 37 ºC (heat) for 3 d. After heat treatment, fruits were transferred to 20 ºC and treated with 10 µl l^–1^ ethylene (C_2_H_4_) for 4 d.

Carotenoid (µg g^–1^ FW)	0 d	3 d		3 d 37 ºC+4 d C_2_H_4_
		20 ºC	37 ºC	
C_40_				
Phytoene	1.13±0.27	2.54±0.10	3.65±0.17	8.28±0.89
Phytofluene	ND	0.65±0.12	0.34±0.06	1.95±0.02
β-Cryptoxanthin	0.17±0.03	0.19±0.03	0.44±0.05	0.26±0.01
Antheraxanthin	2.76±0.03	5.70±0.55	6.61±0.07	11.51±2.50
Zeaxanthin	1.07±0.35	0.62±0.18	0.91±0.12	0.70±0.08
*all-E*-Violaxanthin	5.69±0.71	8.95±0.72	6.30±0.19	5.56±1.07
(9*Z*)-Violaxanthin	26.80±2.46	35.91±3.27	24.28±1.34	29.11±0.30
C_30_				
β-Citraurin	4.76±0.23	6.47±0.21	0.73±0.12	6.78±0.51

The values are means ±SD of at least three measurements.

ND, not detected.

### Functional analysis of Citrus CCD4b1

**Fig. 6.**
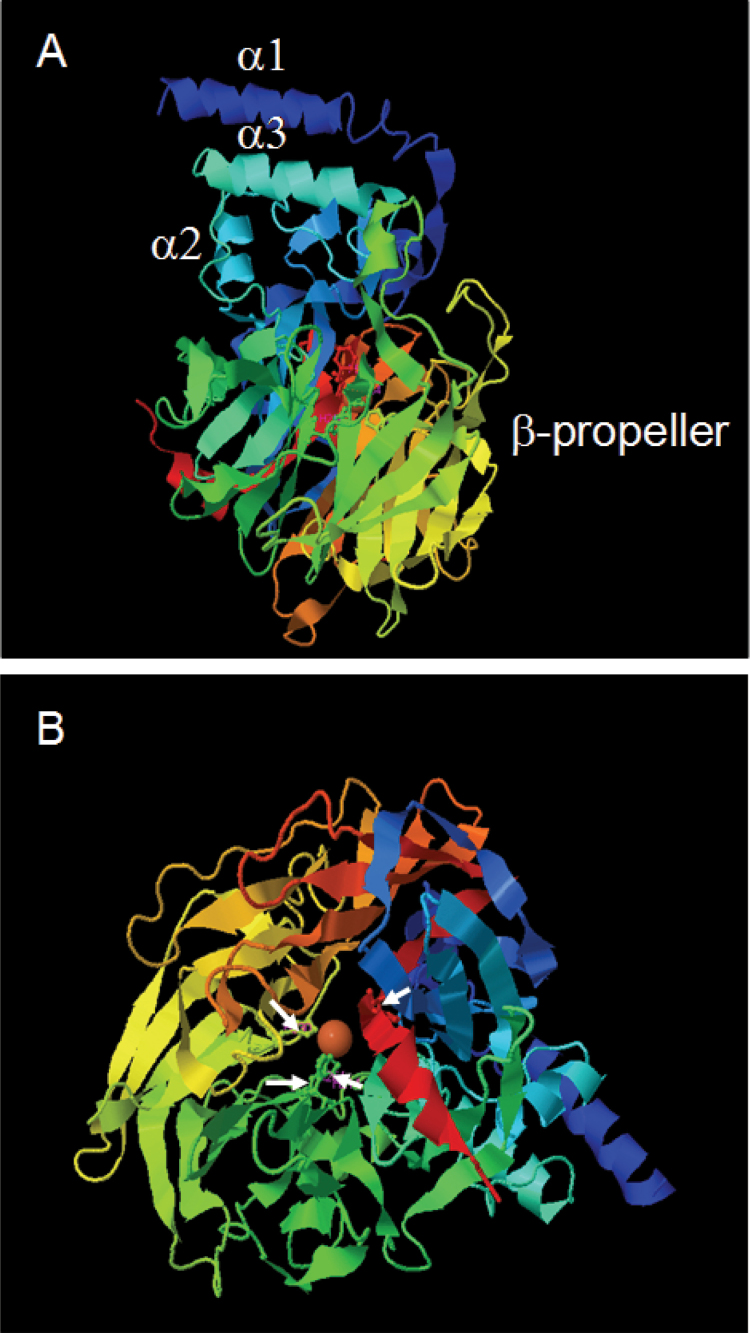
Best 3D model of CCD4b1 using the VP14 (PBD: 2biwA) structure from maize as template. The α-helix (α1, α2, and α3) and β-propeller domains are shown in model view (A). A top view of the model allows the visualization of the propeller blades and the Fe^2+^ in the reaction centre (B). White arrows in (B) indicate the location of the four histidines coordinating the Fe^2+^. (This figure is available in colour at *JXB* online.)

In order to gain insights into the functionality of *Citrus* CCD4b1, the 3D structure was modelled using the I-TASSER online platform (Zhang, 2008; Roy *et al.*, 2010) using two different CCD-type enzyme structures as templates: the apocarotenoid cleavage oxygenase (ACO) from *Synecchocystis* (PBD accession: 2biwA; Kloer *et al.*, 2005) and the 9-*Z*-epoxycarotenoid dioxygenase (VP14) from maize (PBD accession: 3npeA; Messing *et al.*, 2010). The C-score for the best 3D CCD4b1 model, which estimates the quality of predicted models (Zhang, 2008; Roy *et al.*, 2010), was higher when VP14 was used as template (Supplementary Fig. S1 at *JXB* online). It is remarkable that in the predicted CCD4b1 structure the two main functional domains of VP14 are very well conserved; the helical domain formed by two antiparallel α-helices, α1 and α3, and the β-propeller structure which forms a long tunnel on the central axis of the protein where the Fe^2+^ is located (Fig. 6A). The predicted CCD4b1 3D structure allowed the visualization of the four histidines of the catalytic centre coordinating the Fe^2+^ (Fig. 6B).

**Fig. 7.**
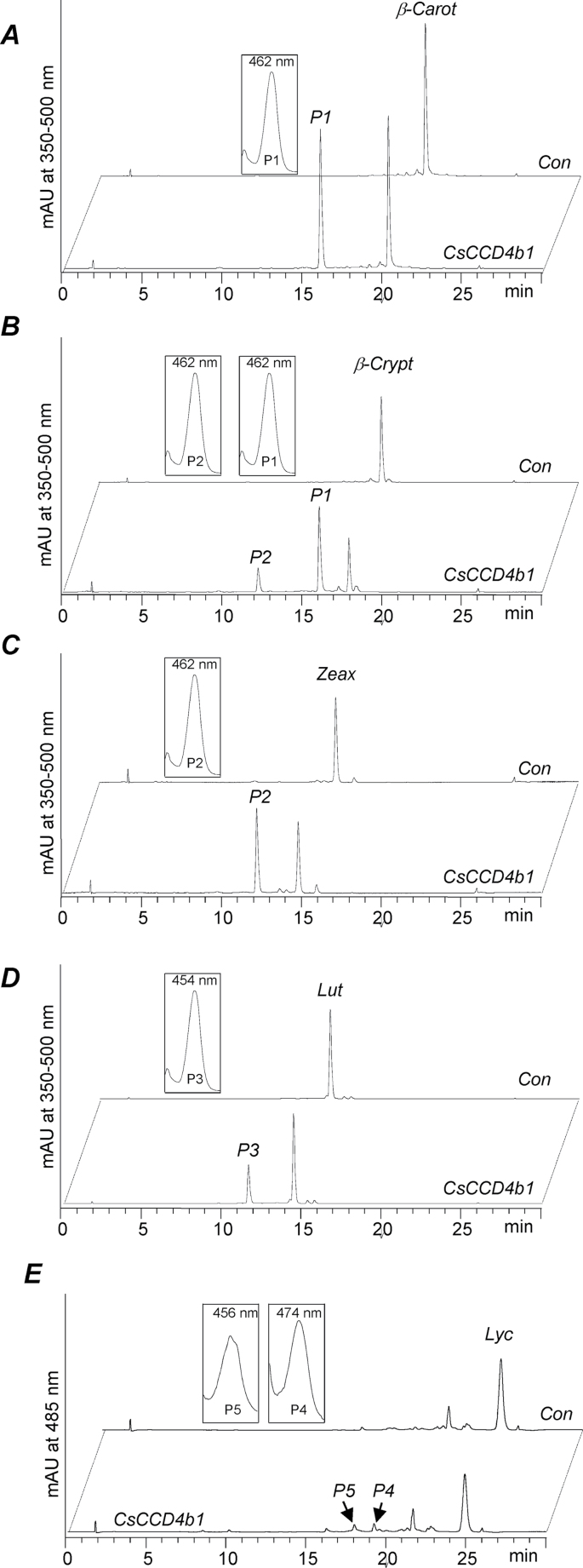
HPLC analysis of the *in vitro* enzymatic activity of *Citrus* CCD4b1. Assays were incubated for 1h, except for lycopene which was incubated for 6h. The crude lysate of thioredoxin-CCD4b1-expressing *E.coli* cells (CCD4b1) converted (A) β-carotene into β-apo-8′-carotenal (P1), (B) β-cryptoxanthin into P1 and β-citraurin (P2), (C) zeaxanthin into P2, (D) lutein into 3-OH-ε-apo-8′-carotenal (α-citraurin) (P3), and (E) lycopene into apo-8′-lycopenal (P4) and apo-10′-lycopenal (P5). UV-Vis spectra of obtained products are depicted in the insets. No conversion was observed with the corresponding controls corresponding to crude lysates of thioredoxin-overexpressing cells (Con).

To investigate the potential cleavage activity of *Citrus* CCD4b1, the full-length cDNA, excluding the first 41 amino acids which are predicted to be the transit plastid peptide, from *C. clementina* was cloned into pBAD-Thio for expression in *E. coli*. The cleavage activity of the crude lysate from the overexpressing cells was assayed using a variety of substrates, and the products were characterized by HPLC-PDA and/or GC-MS. Since the putative *in vivo* precursors of the common C_30_ apocarotenoids are thought to be β-cryptoxanthin, zeaxanthin, and β-carotene, these carotenoids were the first to be tested as substrates of CCC4b1. Assays with β-carotene and zeaxanthin gave a unique product whose retention time and spectrum characteristics were coincident with standards of β-apo-8′-carotenal and β-citraurin, respectively, indicating a cleavage reaction at the 7′,8′ or 7,8 position of the carotenoid backbone (Fig. 7A, C). When β-cryptoxanthin was assayed as substrate, a mixture of two products was identified: β-apo-8′-carotenal and β-citraurin, both resulting from the asymmetric and eccentric cleavage at the 7′,8′or 7,8 double bonds. However, in this case, the preference of the enzyme seemed to be in favour of the production of β-apo-8′-carotenal (cleavage at the 7′,8′ bond on the hydroxylated moiety) since a higher proportion of this apocarotenoid was detected (Fig 7B). Cleavage of the 7′,8′ double bond in β-carotene and zeaxanthin should also lead to further products (C_10_), namely β-cyclocitral and 3-OH-cyclocitral, respectively. Indeed, the enzyme produced β-cyclocitral (C_10_) upon incubation with β-carotene (Supplementary Fig. S2 at *JXB* online). In order to explore the substrate specificity of CCD4b1 further, two additional cyclic carotenoids (α-carotene and lutein) with β- and ε-rings, and one linear carotenoid (lycopene) were also examined. In assays containing α-carotene, one single C_30_ product, ε-apo-8′-carotenal, plus the C_10_ β-cyclocitral were detected (data not shown), while in the assays using lutein, only 3-OH-8′-apo-ε-carotenal (α-citraurin) was identified (Fig. 7D). The results indicated that CCD4b1 was able to cleave carotenoid structures with a ε-ring but only at the 7′,8′ position and not at the 7,8 bond (i.e. only on the moiety containing the β-ionone ring). Interestingly, when lycopene was used as a substrate, the activity was very low and two different apocarotenoids were detected: apo-8′-lycopenal (C_30_) and apo-10′-lycopenal (C_27_) which are predicted products derived from cleavage of lycopene at the 7,8 and 5,6 double bonds, respectively (Fig. 7E). Linear apolycopenals (apo-8′-lycopenal and apo-10′-lycopenal) were also tested as potential substrates, and the C_22_ and C_19_ dialdehydes were detected (Supplementary Fig. S3), indicating a cleavage at the 5,6 double bond.

## Discussion

Pigmentation of *Citrus* fruits is a key quality attribute that is determined by the carotenoid content and composition (Gross, 1987). During the last years, the molecular basis of carotenoid biosynthesis and accumulation has been investigated in depth, and most of the genes involved have been isolated and characterized (Kato *et al.*, 2004; Rodrigo *et al.*, 2004; Alquézar *et al.*, 2008*a*; Kato, 2012). Besides the characteristic C_40_ carotenoids, *Citrus*-specific C_30_ apocarotenoids, mainly β-citraurin and, in a minor proportion, β-apo-8′-carotenal, are major pigment constituents causing the attractive and intense orange coloration of mandarin and orange fruits (Zechmeister and Tuzson, 1936, 1937; Curl, 1965; Stewart and Leuenberger, 1976; Stewart, 1977; Gross, 1981; Oberhoslter *et al*., 2001). The main objective of this work was to identify the enzyme(s) responsible for the synthesis of C_30_ apocarotenoids of *Citrus* peel. To date, many CCD activities have been described in plants, fungi, bacteria, and animals with different centric, eccentric, or symmetric cleavage at specific positions of the carotenoid molecules (reviewed by Auldridge *et al*., 2006; Kloer and Schulz, 2006; Ohmiya, 2009; Walter and Strack, 2011). However, no activity has been reported that converts parent C_40_ carotenoids into C_30_ plus C_10_ apocarotenoids. On the other hand, some C_30_ apocarotenoids or their derivatives, such as β-apo-8′-carotenal, are widely used as food, cosmetic, and pharmaceutical colorants and are obtained from chemical synthesis. Therefore, the identification and characterization of such a CCD enzyme that catalyses this reaction may lead to novel biotechnological strategies for the commercial production of C_30_ apocarotenoids.

Taking advantage of currently available genomic and transcriptomic data on *Citrus* sp., the databases were screened searching for novel *CCD* genes with similarity to saffron ZCD (Bouvier *et al.*, 2003). Among the CCD activities reported in plants, the reaction catalysed by ZCD is the most similar to that predicted for the *Citrus* enzyme(s) involved in C_30_ biosynthesis, although the *Citrus* enzyme should cleave eccentrically only on the 7′,8′ or 7,8 position to render C_30_ and C_10_ apocarotenoids. The search resulted in the identification of a new *CCD4* family of five genes (Table 1). Two of these genes, *CCD4a* and *CCD4b1*, were previously identified by Agustí *et al*. (2007) in an EST collection from cDNA libraries from Clementine mandarin, and the other three, *CCD4b2*, *CCD4c*, and *CCD4d*, had not been previously reported. The sequences of the predicted proteins were almost identical in orange and mandarin, except for CCD4b2 and CCD4d. Interestingly, *CCD4b2* and *CCD4d* genes are located on the same chromosomes as *CCD4b1* and *CCD4c*, respectively (Table 1). The presence of tandem gene duplications is a common feature of the *CCD4* gene family in many different plants species, resulting in the generation of high identity and/or truncated copies of the original gene (Ahrazem *et al.*, 2010). It is thus likely that in the *Citrus* genomes a similar mechanism may have resulted in the *CCD4b2* and *CCD4d* genes originating from *CCD4b1* and *CCD4c*, respectively. The duplicated *CCD4b2* and *CCD4d* genes in sweet orange and mandarin could then accumulate mutations due to less evolutionary pressure. This would explain the higher sequence variability existing between the orthologues of these genes in these two *Citrus* species.

Accumulation of C_30_ apocarotenoids in *Citrus* is fruit specific and appears to be developmentally regulated. It has been described that the presence of C_30_ apocarotenoids is almost restricted to the peel of ripe *Citrus* fruit (Gross, 1987), and it is likely that the genes involved in their biosynthesis may be stimulated during the process and enhanced in this tissue. Analysis of *Citrus* EST collections revealed that *CCD4b1* ESTs were more highly represented in libraries obtained from fruit tissues, while ESTs of *CCD4a* and *CCD4c* were identified in libraries made of whole-plant tissues or mixed tissues. A detailed expression analysis of the *CCD4-like* genes in different vegetative and reproductive organs showed contrasting patterns (Fig. 3). Interestingly, *CCD4a*, *b1*, and *c* were expressed in petals, which is consistent with the fact that in other plant species *CCD4* genes are preferentially expressed in flower organs (Ohmiya *et al.*, 2006; Huang *et al*., 2009; Rubio *et al*., 2009; Yamamizo *et al.*, 2010; Yoshioka *et al.*, 2012). In chrysanthemum flowers, white petal coloration is linked to the presence of CCD4a activity, which catabolizes coloured carotenoids (Ohmiya *et al.*, 2006). In petals of orange, the expression of some carotenoid biosynthetic genes has been reported (Alquézar *et al.*, 2009), suggesting that carotenoid biosynthesis is active, although this tissue is white and devoid of carotenoids. Therefore, it is tempting to speculate that the activity of CCD4, particularly CCD4c that is restricted to petals, may be related to the cleavage of carotenoids to colourless apocarotenoids and C_9_ to C_13_ volatile apocarotenoids (norisoprenoids) which are potent aroma compounds. *CCD4a* transcripts were detected in all *Citrus* organs analysed, but the relative expression was higher in leaves (Fig. 3), and in fruit peel showed a pattern not consistent with that of C_30_ apocarotenoid accumulation (Supplementary Fig. S4 at *JXB* online). Furthermore, a role related to carotenoid turnover in leaves has also been proposed for some CCD4s (Walter and Strack, 2011). Interestingly, the expression of *CCD4b1* was almost exclusively detected in peel of ripe fruit (Fig. 3). Also this gene is more distantly related to the other *CCD4-like* genes since is located in a separate cluster in the phylogenetic tree (Fig. 2). In the flesh of peach fruit, high transcript levels of a *CCD4* gene have been correlated with the production of volatile apocarotenoids and degradation of carotenoids (Brandi *et al*., 2010); however, the functional characterization of this protein has not been described yet.

Accumulation of C_30_ apocarotenoids, mainly β-citraurin, in the peel of ripe fruits clearly correlates with colour intensity; however, there is little information about the changes in C_30_ apocarotenoids during *Citrus* fruit development and ripening. In fruits of the three *Citrus* genotypes studied, C_30_ apocarotenoids were not detected in green fruits, but the concentration of β-citraurin increased substantially from breaker to fully coloured fruits (Fig. 4). Different lines of evidence indicate a casual relationship between the transcript level of *CCD4b1* and the β-citraurin content: (i) expression of the gene and the presence of the apocarotenoid were exclusively restricted to fruit peel; and (ii) peel of the *Citrus* genotype with a higher content of β-citraurin (cv. Fortune hybrid) also showed higher expression levels of *CC4Db1* (Fig. 4). Moreover, no expression of the *CCD4b* gene was detected in fruit of a Clementine mandarin mutant with a pale yellow external colour which is devoid of β-citraurin (Ríos *et al.*, 2010; unpublished results). Maximum accumulation of β-citraurin was not fully coincident with the highest expression level of *CCD4b1* (breaker or coloured stage, Fig. 4). This may indicate that the availability or accessibility of the precursors may be a further factor governing β-citraurin content. To explore this hypothesis, the concentrations of the substrates predicted for the cleavage reaction (i.e. zeaxanthin and β-cryptoxanthin; Farin *et al.*, 1983) were determined. Accumulation of β-cryptoxanthin was fairly parallel to that of β-citraurin in the peel of the three genotypes studied, suggesting that this xanthophyll is the most likely *in vivo* precursor of this apocarotenoid. Nevertheless, the presence of zeaxanthin in the peel of the samples analysed (Fig. 4), especially Clementine mandarin and sweet oranges, does not allow this xanthophyll to be dismissed as a precursor.

Application of ethylene triggers peel pigmentation by stimulating both chlorophyll degradation and the accumulation of carotenoids, including the apocarotenoid β-citraurin (Stewart and Wheaton, 1972; Rodrigo and Zacarías, 2007). In fruits of sweet orange and Clementine mandarin, it was found that ethylene-induced enhancement of external colour (Fig. 5) was associated with an increase in β-citraurin content and stimulation (mandarin) or maintenance (sweet orange) of *CCD4b1* transcript levels (Table 2, Fig. 5). Ethylene treatment also increased the concentration of other carotenoids such as β-cryptoxanthin or phytoene, in agreement with previous data (Stewart and Wheaton, 1972; Rodrigo and Zacarías, 2007; Matsumoto *et al.*, 2009).

On the other hand, it is known that *Citrus* fruits grown in the tropics, where the temperature is >30 ºC, develop poor colour (Stewart and Wheaton, 1972). Thus, a heat conditioning treatment was selected for its effect on decreasing external coloration (Plaza *et al.*, 2004) (Fig. 5B). This loss of peel pigmentation was associated with a dramatic decrease in β-citraurin and the suppression of *CCD4b1* expression (Table 3, Fig. 5B). A similar effect was also observed in experiments performed with mandarins (data not shown). Interestingly, the treatment of previously heated fruits with ethylene was able to recover *CCD4b1* gene expression and β-citraurin content (Table 3, Fig. 5B). Storage of Satsuma mandarin fruits at 30 ºC has been shown to increase zeaxanthin and β-carotene content, but, unfortunately, no data on β-citraurin or fruit colour were reported (Matsumoto *et al.*, 2009). The repression of *Citrus CCD4b1* at high temperatures contradicts the reported heat-mediated induction of other plant *CCD4* genes whose expression is enhanced by heat stress (Rubio *et al.*, 2008). This contrasting pattern of *CCD4b1* expression in *Citrus* may be due to its different function and its involvement in other physiological processes.

Previous studies on carotenoid biosynthesis in orange-coloured *Citrus* fruits of different varieties have shown a coordinated regulation for most of the genes to produce the main chromoplastic carotenoids: 9-*Z*-violaxanthin and β-cryptoxanthin. Interestingly, key genes regulating carotenoid accumulation in *Citrus* fruits, such as *PSY*, *β-LCY2*, and *β-CHX*, displayed a transcriptional pattern very similar to that described in the current study for *CCD4b1* during natural ripening (Kato *et al.*, 2004; Rodrigo *et al.*, 2004; Alquézar *et al.*, 2008*b*, 2009) and also in response to treatments enhancing fruit colour (Fujii *et al.*, 2007; Rodrigo and Zacarias, 2007; Matsumoto *et al.*, 2009; Carmona *et al.*, 2012). These observations may indicate that the *CCD4b1* gene is transcriptionally regulated during fruit ripening in a pattern similar to that of other key genes of carotenoid biosynthesis and that the global flux of the pathway is coordinately modulated to produce the end-products accumulating in the peel of mature fruits, such as β,β-xanthophylls and C_30_ apocarotenoids.

The crystallographic structures of two CCD enzymes, ACO from the cyanobacteria *Synechocystis* and VP14 from maize, have revealed important functional domains and also facilitated the identification of residues involved in the catalytic centre or the substrate specificity (Kloer *et al.*, 2005; Messing *et al.*, 2010). In order to identify putative structural 3D domains in CCD4b1, a tertiary structure was modelled using ACO and VP14 as templates. The 3D CCD4b1 model was better adjusted to the VP14 structure (Supplementary Fig. S1 at *JXB* online) and the α-helical domain, which is predicted to interact with plastid membranes, and the β-propeller structure containing the catalytic centre, were identified (Fig. 6). Unfortunately, the dome domain or the tunnel entrance to the reaction centre could not be well defined by the modelling since these positions display high variability (Kloer *et al.*, 2005). Nevertheless, alignment of the CCD4b1 sequence with VP14 indicates conserved amino acid residues in positions important for substrate or cleavage specificity such as Phe171 or Val478 in VP14, corresponding to Phe131 and Leu433 in CCD4b1 (data not shown).

A further challenge in this study was to demonstrate the cleavage activity of CCD4b1 and to investigate its substrate specificity. To that end, the protein was overexpressed and the crude lysate was assayed *in vitro*. Several cyclic β,β-ring carotenoids were tested, including the most likely *in vivo* precursors, such as β-cryptoxanthin and zeaxanthin. In addition, β-carotene was also assayed since in some *Citrus* species, such as mandarins, the presence of small amounts of β-apo-8′-carotenal has been reported (Curl, 1965; this study). Since β-carotene and zeaxanthin are symmetrical molecules, only a single product was detected, corresponding to β-apo-8′-carotenal and β-citraurin, respectively (Fig. 7). However, for β-cryptoxanthin, the two possible C_30_ apocarotenoids were obtained: β-apo-8′-carotenal and β-citraurin, although their relative abundance may indicate that the reaction was in favour of β-apo-8′-carotenal formation. Moreover, the presence of the volatile C_10_ β-cyclocitral was also detected in the assays using β-carotene and β-cryptoxanthin as substrates (Supplementary Fig. S2 at *JXB* online). Based on the *in vitro* results, it can be concluded that both xanthophylls, β-cryptoxanthin and zeaxanthin, are good *in vivo* substrates for CCD4b1, and the availability or accessibility to the enzyme would determine the specificity. Interestingly, minor amounts of β-apo-8′-carotenal have been also reported in *Citrus* peel, which is consistent with CCD4b1 activity on β-cryptoxanthin in coloured fruits, or on β-carotene in fruits at the breaker stage (Table 2). To test the influence of the ionone end-ring structure or the linear backbone on the cleavage activity, α-carotene, lutein, and lycopene were assayed. Interestingly, C_30_ apocarotenoids were generated in the α-carotene and lutein assays but the cleavage was at the side of the β-ionone ring, indicating a clear preference for that position. The activity for lycopene was very low, but the presence of C_30_ apo-8′-lycopenal was also detected (Fig. 7). However, in the reactions performed with acyclic carotenoids, a small proportion of an apo-10′-lycopenal C_27_ was also identified, indicating a cleavage at the 5,6 position. Moreover, when apolycopenals were used as substrates, cleavage of the 5,6 double bond was also detected (Supplementary Fig. S3). This may indicate that the absence of the ionone ring can substantially alter not only the activity, but also the cleavage position specificity. The resulting C_30_ products from the cleavage of lutein, α-carotene, and lycopene were not reported in *Citrus* extracts, although lutein and, in a minor proportion, α-carotene, are typical carotenoids in green fruits (Gross, 1987; Kato *et al.*, 2004; Rodrigo *et al.*, 2004). This might indicate that *in planta* these carotenoids are not substrates for the enzyme, or the activity of CCD4b1 enzyme in green fruits is too low for an efficient cleavage.

To the authors’ knowledge this is the first report of an eccentric CCD cleavage activity on a C_40_ carotenoid at the 7,8 or 7′,8′ double bonds. The CCD/CCO enzymes reported to date have a broad spectrum of cleavage positions and/or substrate specificities (reviewed in Walter and Strack, 2011). Some of them appear to have a more general and ubiquitous presence in plants, such as members of the CCD1, NCED, CCD7, or CCD8 families, which are involved in the generation of signalling molecules and phytohormones (Tan *et al.*, 1997; Schwartz *et al.*, 2004; Vogel *et al.*, 2010; Kohlen *et al.*, 2012). Others, such as some members of the CCD1 or the CCD4 subfamily, may have specialized functions in specific organs, such as the production of scent and aroma compounds in flowers and other reproductive tissues or a function more related to carotenoid turnover under stress responses (Ahrazem *et al.*, 2010; Walter *et al.*, 2010). The *Citrus CCD4-like* family is composed of five members that probably originated by duplications and divergence of an ancestral gene, giving rise to the diversity in expression patterns and, probably, in function. Thus, accumulation of mutations in a duplicated gene from the ancestral *CCD4* without selection pressure might be the evolutionary origin of the specialized novel cleavage activity of *Citrus* CCD4b1 restricted to fruit peel tissue. The agronomical selection of orange and mandarin cultivars with an intense orange-red peel coloration, and hence high CCD4b1 activity and high concentration of C_30_ reddish apocarotenoids in the peel, have contributed throughout the years to maintain and propagate this quality trait.

## Supplementary data

Supplementary data are available at *JXB* online.

Figure S1. Best 3D models of *Citrus* CCD4b1 obtained with the I-TASSER online platform by using as templates the 3D structure of CCO (PBD: 2biwA) from *Synechocystis* (A) and VP14 (PBD: 3npeA) from maize (B).

Figure S2. GC-MS analysis of *in vitro* enzymatic activity of *Citrus* CCD4b1.

Figure S3. *In vitro* CCD4b1 enzymatic assays using apolycopenals as subtrates (A) apo-8′-lycopenal (C_30_); (B) apo-10′-lycopenal (C_27_).

Figure S4. Relative transcript levels of *CCD4a* in the peel of Navel sweet orange, Clementine mandarin, and the hybrid Fortune mandarin during fruit development and ripening.

Table S1. Matrix of similarity of *Citrus* CCD4-like family proteins.

Supplementary Data
