# Supplementary material for: A novel carotenoid cleavage activity involved in the biosynthesis of Citrus fruit-specific apocarotenoid pigments
Source: J Exp Bot. 2013 Sep 4;64(14):4461–78. doi: 10.1093/jxb/ert260 (PMC3808326; doi:10.1093/jxb/ert260)
Supplement: Supplementary Data [file supp_64_14_4461__index.html]

A novel carotenoid cleavage activity involved in the biosynthesis of Citrus fruit-specific apocarotenoid pigments — A novel carotenoid cleavage activity involved in the biosynthesis of Citrus fruit-specific apocarotenoid pigments — Supplementary Data 

# A novel carotenoid cleavage activity involved in the biosynthesis of *Citrus* fruit-specific apocarotenoid pigments

## Supplementary Data

Data files

**Files in this Data Supplement:**

- Supplementary Data - Supplementary Data
