# Supplementary material for: A novel carotenoid cleavage activity involved in the biosynthesis of Citrus fruit-specific apocarotenoid pigments
Source: J Exp Bot. 2013 Sep 4;64(14):4461–78. doi: 10.1093/jxb/ert260 (PMC3808326; doi:10.1093/jxb/ert260)

**Supplemental table S1**. Matrix of similarity between the different *Citrus clementina* CCD4-like family members based on deduced amino acid and sequences alignment (%).

| | CCD4a | CCD4b1 | CCD4b2 | CCD4c |
|---|---|---|---|---|
| CCD4a | 100 | | | |
| CCD4b1 | 48 | 100 | | |
| CCD4b2 | 48 | 88 | 100 | |
| CCD4c | 60 | 45 | 45 | 100 |
| CCD4d | 39 | 32 | 30 | 49 |

**Supplemental Figure S1**. Best 3D models of *Citrus* CCD4b1 by using I-TASSER online platform by using as templates the 3D structure of CCO (PBD: 2biwA) from *Synechocystis* (A) and VP14 (PBD: 3npeA) from maize (B) which are showed overlapped in purple ribbons. The C-score obtained for each model is indicated below the images. C-score is a confidence score for estimating the quality of predicted models by I-TASSER. It is calculated based on the significance of threading template alignments and the convergence parameters of the structure assembly simulations. C-score is typically in the range of [-5,2], where a C-score of higher value signifies a model with a high confidence and vice-versa.

Zhang Y. (2008). I-TASSER server for protein 3D structure prediction. BMC Bioinformatics. 9: 40;. Roy A., Kucukural A. and Zhang Y.(2010). I-TASSER: a unified platform for automated protein structure and function prediction. Nature Protocols 5: 725-738.

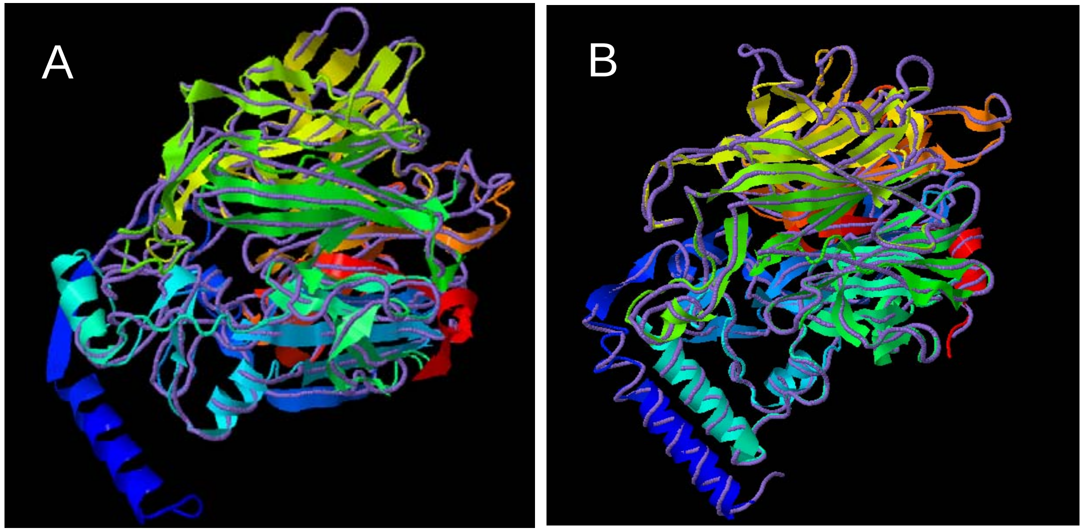


C-score 0.77 C-score 1.96

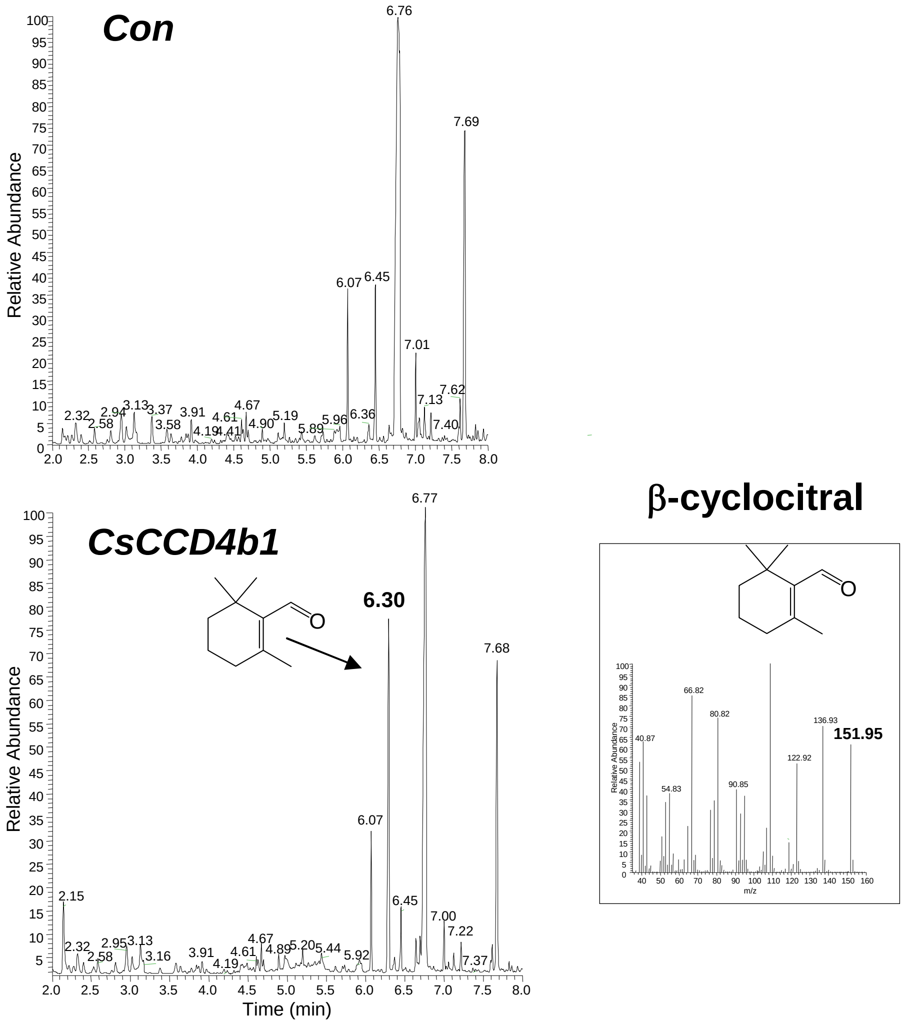


**Suplemental Figure S2**. GC-MS analysis of *in vitro* enzymatic activity of citrus CCD4b1. Gas phase of *in vitro* assays performed with β-carotene or β-cryptoxanthin and crude lysate of thioredoxin-CCD4b1 expressing *E. coli* cells (CCD4b1) and control lysate obtained from thioredoxin expressing cells (Con) was extracted using an open loop and applied to GC-MS analysis. The total ion current revealed a new compound formed by thioredoxin-CCD4b1, which eluted at min 6.30. Mass spectrum of this product identified it as β-cyclocitral.

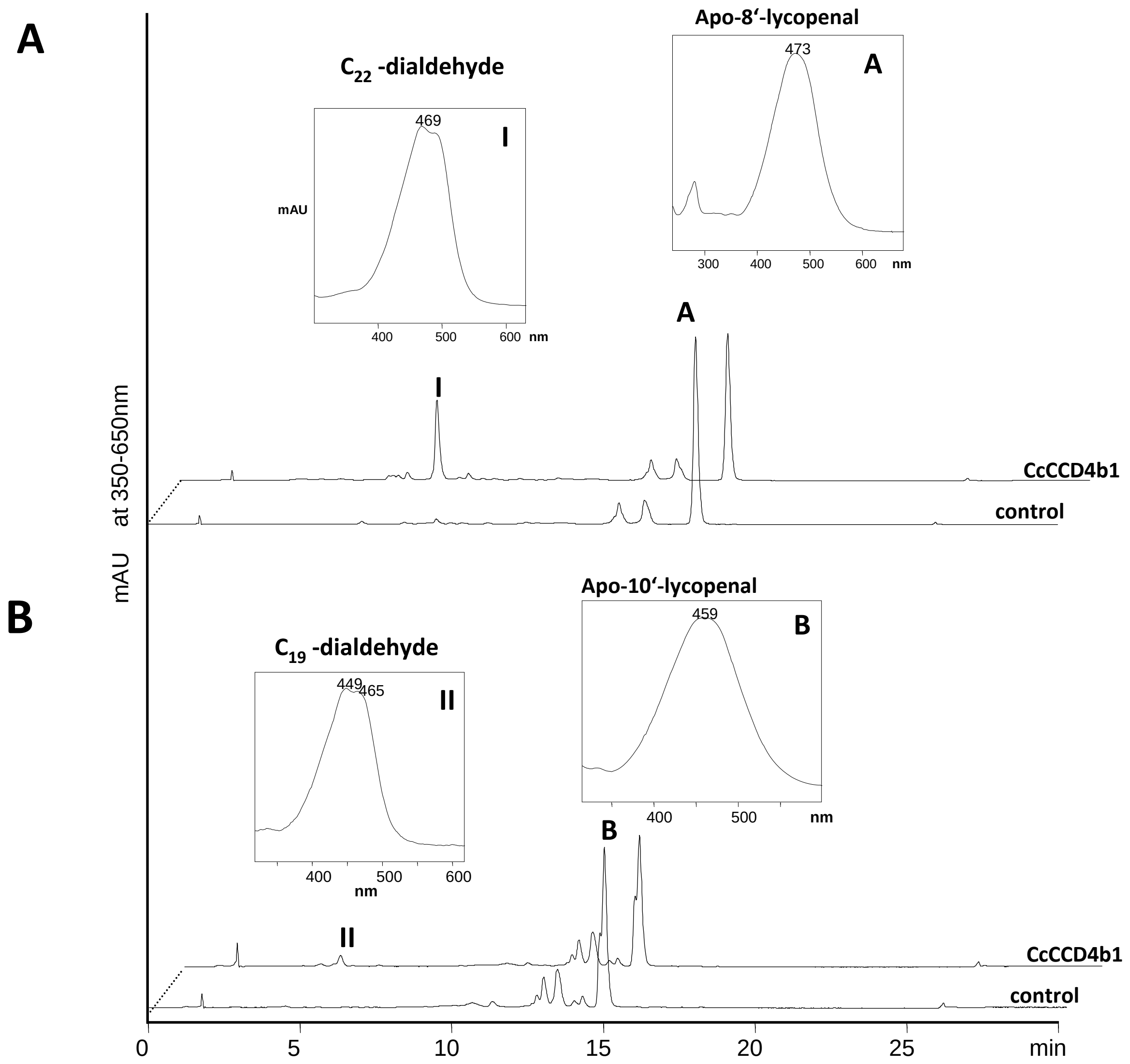


**Supplemental Figure S3.** *In vitro* CCD4b1 enzymatic assays using apo-lycopenals as subtrates (A, Apo-8´-lycopenal ($C_{30}$); B apo-10´-lycopenal ($C_{27}$).

**Figure S4.** Relative transcript levels of *CCD4a* in the peel of Navel sweet orange, Clementine mandarin and the hybrid Fortune mandarin during fruit development and ripening.

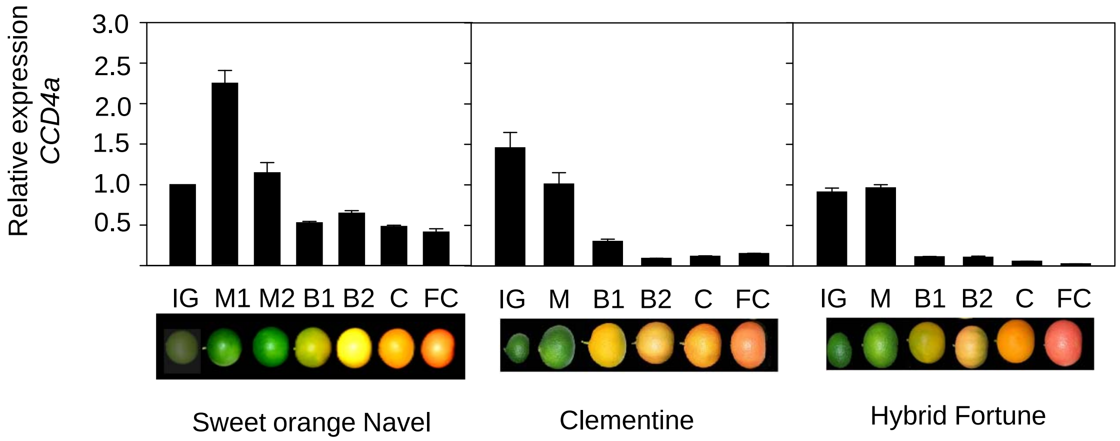

Supplement: Supplementary Data [file supp_ert260_jexbot100453_file001.pdf]
